# Metal–Ligand Cooperation in Dihydrogen Activation by a Cationic Metallogermylene: Enhanced Activity from Tungsten to Molybdenum

**DOI:** 10.3390/molecules29245974

**Published:** 2024-12-18

**Authors:** Rikiya Matsumoto, Koichi Nagata, Ryo Nakamura, Takahito Watanabe, Hisako Hashimoto

**Affiliations:** Department of Chemistry, Graduate School of Science, Tohoku University, 6-3 Aramaki, Aoba-ku, Sendai 980-8578, Japan; cherry.s5085@gmail.com (R.M.); koichi.nagata.d3@tohoku.ac.jp (K.N.); ryo.nakamura.t8@dc.tohoku.ac.jp (R.N.); takahito.watanabe@gmail.com (T.W.)

**Keywords:** metallotetrylene, germylene, dihydrogen activation, metal–ligand cooperation

## Abstract

Dihydrogen activation by metallogermylenes was investigated experimentally and theoretically. A neutral NHC-coordinated chlorometallogermylene was synthesized and converted to a cationic base-free metallogermylene of molybdenum via chloride abstraction. The cationic molybdogermylene showed enhanced reactivity toward H_2_ compared to the tungsten analog. The reaction mechanism was investigated by theoretical calculations, which revealed a novel route that proceeds via a new type of metal–ligand cooperative activation between the metal and divalent germanium moiety. The activation energy of this route is much lower than that of the alternative route via an “oxidative addition” type of reaction on the single Ge(II) center, which is generally proposed for organotetrylenes. The features of the frontier orbitals and the origin of the metal effect on the H_2_ activation are also described.

## 1. Introduction

Dihydrogen (H_2_) is the simplest molecule, but its potential for applications can be the highest in current chemistry. Activation of the H–H σ bond of molecular dihydrogen is the key to unlocking the full potential of dihydrogen and has been extensively studied [1,2,3]. The development of efficient and sustainable strategies that can promote the H–H bond activation is highly appreciated from the aspect of synthetic chemistry as well as energy storage issues and sustainable chemistry [4,5,6,7]. The classical dihydrogen activation relies on a single active site of transition-metal (TM) complexes, i.e., oxidative addition that proceeds via σ-donation from H_2_ to the coordinatively unsaturated metal and π-back donation from the metal d-electron to the σ* (H–H) orbital [8,9]. In recent years, metal–ligand cooperative reactions, in which a metal and a ligand participate in the cleavage of the H–H bond, have emerged as new strategies. These include (i) metal hydrogenation via protonation (or hydridation), whereby the ligand acts as a Lewis acid (or base), (ii) metal hydrogenation driven by the aromatization and dearomatization of the *N*-heterocyclic ligand, (iii) metal hydrogenation involving hydrogen addition to the carbene (tetrylene), and (iv) H–H bond polarization and cleavage between metals in bimetallic complexes [10,11,12,13,14,15,16,17,18,19,20,21].

**Figure 1:**
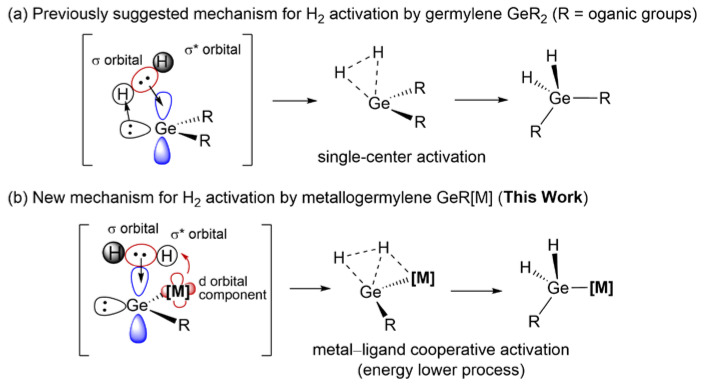
Schematic description of (**a**) previously suggested mechanism for H_2_ activation by germylene GeR_2_ (R = organic substituents); (**b**) new mechanism for H_2_ activation by metallogermylene GeR[M] (this work).

There are also examples of activation of small molecules by heavier divalent group-14 species (tetrylenes) [22,23], which contain a lone pair of electrons and a vacant p orbital on the group-14 element [24]. In these cases, theoretical calculations have shown that activation of H_2_ is promoted by synergistic interactions between the lone-pair orbital on the tetrylene and the σ* orbital of dihydrogen as well as the σ-bonding orbital of dihydrogen and the empty p orbital of the tetrylene, mimic to “oxidative addition” on TM (Figure 1a). As relevant compounds, metal-substituted tetrylenes, i.e., metallotetrylenes, have been synthesized, and their properties have started to be clarified, albeit with limited examples [25,26,27,28,29].

**Scheme 1:**
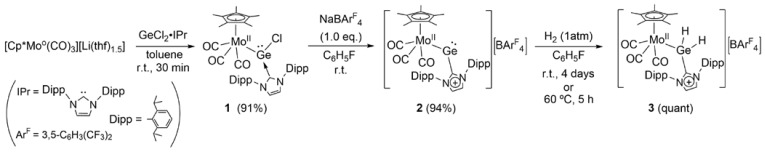
Synthesis of molybdogermylenes (**1** and **2**) and the activation of H_2_ by **2**.

In this regard, our group has previously succeeded in synthesizing a cationic metallogermylene of tungsten, [Cp*W(CO)_3_–Ge(IPr)] [BAr^F^_4_] [**2-W**; Ar^F^ = 3,5-C_6_H_3_(CF_3_)_2_], by the chloride abstraction of an *N*-heterocyclic-carbene (NHC)-coordinated chlorometallogermylene [30] (see Scheme 1: **2-W** is a tungsten analog of **2**, vide infra). This complex activated the σ bond of dihydrogen, hydrosilanes, and hydroboranes, representing the first example of the successful activation of small molecules by a metallogermylene [31]. As our continued interest in this system, we synthesized the molybdenum analog of **2-W**, [Cp*(CO)_3_Mo–Ge(IPr)][BAr^F^_4_] (**2**) to investigate the influence of the central metal in dihydrogen activation. As a result, we found a new lower energy pathway that involves a significant contribution of d-orbitals, leading to a new type of metal–ligand cooperation, as illustrated in Figure 1b. Herein, we report the synthesis of metallogermylenes of molybdenum and the details of dihydrogen activation by the cationic metallogermylene.

## 2. Results and Discussion

Cationic metallogermylene of molybdenum **2** was successfully obtained in two steps by applying the method for **2-W** (Scheme 1). Initially, a precursor neutral complex Cp*(CO)_3_Mo–GeCl(IPr) (**1**) was prepared in 91% yield as a yellow powder by the desalting reaction of [Cp*(CO)_3_Mo][Li(thf)_1.5_] with GeCl_2_(IPr). Subsequent chloride abstraction from **1** with Na[BAr^F^_4_] in fluorobenzene at room temperature furnished **2** in the form of air- and moisture-sensitive yellow-green crystals. Complex **2** was dissolved in C_6_H_5_F and *o*-DCB-*d*_4_, allowing us to collect NMR data in solution (Figures S7–S11), in contrast to **2-W** which became almost insoluble after being crystallized. Spectroscopic analyses (NMR and FT-IR) and a single-crystal X-ray diffraction analysis (SC-XRD) suggest that **1** and **2** are neutral-base-stabilized and cationic-base-free metallogermylenes, respectively. In the ^1^H NMR spectrum of **1** in C_6_D_6_, signals for the *i*-Pr groups of the IPr ligand appear as four sets of a doublet for CH_3_ (at 0.98, 0.99, 1.54, and 1.66 ppm) and two sets of a septet for CH (at 3.07 and 3.18 ppm), indicating the diastereotopic feature, similarly to tungsten complex **1-W**. In contrast, the ^1^H NMR spectrum *(o*-DCB-*d*_4_) of **2** exhibited two sets of a doublet for CH_3_ and a single septet for CH for the *i*-Pr groups, indicating that four *i*-Pr groups in **2** are magnetically equivalent. These results agree that the germanium center in **2** is achiral in solution.

**Figure 2:**
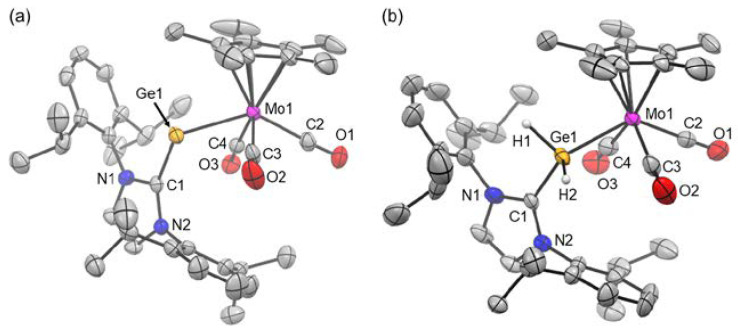
Molecular structures of (**a**) cationic molybdogermylene **2** and (**b**) dihydrogermyl complex **3** in the crystalline state with thermal ellipsoids at 50% probability (one of two independent molecules is shown for each). Hydrogen atoms bound to carbon atoms and the counter anion [BAr^F^_4_]^−^ are omitted for clarity (for details, see the Supplementary Materials). Selected bond lengths [Å] and bond angles [^o^]. For **2**: Mo1–Ge1 = 2.5701(6), Ge1–C1 = 2.048(4); Mo1–Ge1–C1 = 112.45(11). For **3**: Mo1–Ge1 = 2.6065(6), Ge1–C1 = 1.996(4), Ge1–H1 = 1.50(5), Ge1–H2 = 1.61(5); Mo1–Ge1–C1 = 120.94(13).

The molecular structure of **2** was determined by SC-XRD study with the support of DFT calculations (Figure S21), although there are non-avoidable disordering moieties at Cp* and the counter anion (at CF_3_) (see Section 3.4). The cationic part of one of two crystallographic independent molecules of **2** per unit cell is shown in Figure 2. The germanium atom of **2** is two-coordinated and bound to the molybdenum atom with Mo1–Ge1–C1 angles of av. 112.7° (Figure 2a). The bent structure around the germanium atom is one of the characteristics of base-free germylenes, which is clearly different from the linear structure for the relevant two-coordinated complexes with M≡Ge triple bonds, i.e., germylyne complexes (L*_n_*M≡Ge–R) [26]. The Mo–Ge bond distance [av. 2.5707(6) Å] is almost at the middle of the reported range for Mo–Ge single bonds [2.27–3.07 Å] [32]. A comparison of these values with those of tungsten analog **2-W** indicates that there is negligible difference in the structural parameters [W–Ge–C: av. 112.4(2)°; W–Ge: av. 2.5788(10) Å] [30].

**Figure 3:**
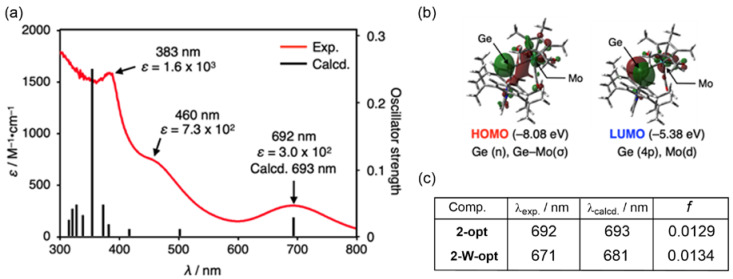
(**a**) UV–vis absorption spectrum (red line) of **2** (1.5 × 10^−2^ M) in C_6_H_5_F (293 K), together with the theoretically calculated absorption for **2-opt** (black bars). (**b**) HOMO and LUMO of **2-opt**. (**c**) Calculated absorption maxima of **2-opt** and **2-W-opt** at the longest wavenumbers. The “*f*” means oscillator strength.

The UV–vis absorption spectrum of **2** provided further insight into its electronic properties in solution (Figure 3a–c). Complex **2** exhibits a weak absorption band with a maximum (*λ*_max_) at 692 nm (*ε* = 3.0 × 10^2^ M^−1^ cm^−1^), a shoulder band at ~460 nm (*ε* = 7.3 × 10^2^ M^−1^ cm^−1^), and an intensive band with a maximum at 383 nm (*ε* = 1.6 × 10^3^ M^−1^ cm^−1^). These absorption bands were examined by TD-DFT calculations on the cationic part of the optimized structure of **2** (**2-opt**) [calcd. Mo–Ge: 2.613 Å; Mo–Ge–C(IPr): 113.05°] (Figure S21). The longest absorption at 692 nm can be ascribed to the HOMO→LUMO (calcd. 693 nm) transition (Table S4). This is essentially the Ge(n)→Ge(4p) transition but has a significant contribution from the d-orbitals of Mo because both HOMO and LUMO contain d-orbital components (Figure 3b). Compared to that of **2-W** (*λ*_max_ = 671 nm) calculated at the same level, the absorption maximum of **2** is bathochromically shifted by ca. 20 nm, indicating that the HOMO-LUMO gap of **2** becomes slightly narrower than that of **2-W** through lowering their energy levels (Figure S23). The dihydrogen activation ability thus can be reinforced by changing **2-W** to molybdogermylene **2**.

The reaction of cationic molybdogermylene **2** with dihydrogen (1 atm) was examined and compared with that of the previously reported tungsten system. The reaction of **2** was completed after 4 days at room temperature or after 5 h at 60 °C, yielding dihydrogermyl complex **3** quantitatively (by ^1^H NMR) (Scheme 1), which proceeded much faster than that of tungsten analog **2-W** with dihydrogen (ca. 14 days at room temperature or 24 h at 60 °C). This comparison clearly shows a significant acceleration of dihydrogen activation by changing the metal from W to Mo. The signal pattern in the ^1^H NMR spectrum of **3** (Figure S13) is almost the same as that of tungsten analog **3-W** [31]. The presence of GeH_2_ is evidenced by a singlet signal that appeared at 3.94 ppm (**2-W:** 3.78 ppm). The structure of **3** was further confirmed by SC-XRD analysis using yellow crystals obtained by recrystallization. Although there was severe disordering in one independent molecule and the counter anion, the whole structure of **3** (Figure S20) and its DFT-optimized structure resemble those of **3-W** (Figure S21). The Mo–Ge bond in **3** [av. 2.6082(6) Å] corresponds to a single bond and is slightly elongated compared with that in **2** [av. 2.57055(7) Å], reflecting the sp^3^-hybridized nature of the germanium center in **3** unlike **2**. A similar change in bond lengths has also been reported for the tungsten system [W–Ge: 2.58 Å (**2-W**); 2.60 Å (**3-W**)] [30]. Accordingly, the Mo–Ge–C bond angle (av.121°) in **3** is wider than that in **2** (av. 111°).

**Figure 4:**
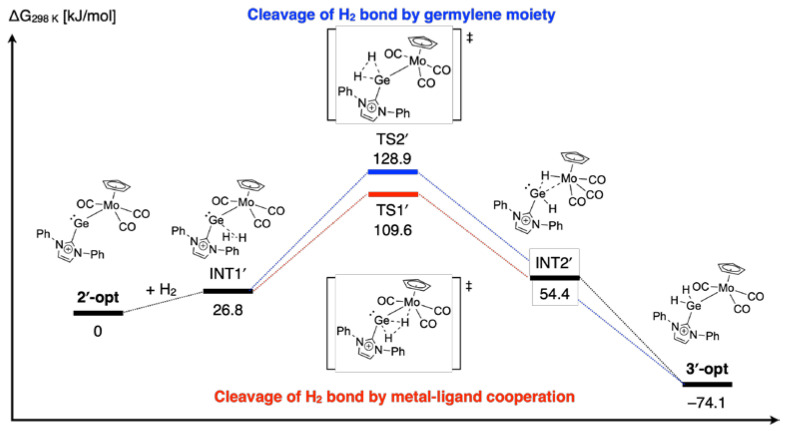
Gibbs free energy profile for the dihydrogen activation using cationic molybdogermylene **2′-opt**, calculated at the M06/LANL2DZ[Mo]:6-31+G(d,p)[H, C, N, O, Ge] (SMD: C_6_H_5_F) level. “‡” indicates a transition state.

To clarify the origin of the observed difference in reactivity toward H_2_ between two metals, we performed theoretical calculations employing model and real complexes (Chart S1, Figure S21). Initially, using model complex **2′-opt**, we found two routes. The Gibbs free energy profiles for the overall reactions at 298 K are illustrated in Figure 4. The reaction starts from the coordination of dihydrogen to the vacant 4p orbital on the germanium atom in **2′-opt**, leading to the formation of adduct **INT1′**. Then, dihydrogermyl complex **3′-opt** forms through either transition state **TS1′** or **TS2′**. Remarkably, the lower route includes **TS1′**, in which one hydrogen atom (H1) approaches germanium, and the other hydrogen (H2) moves toward molybdenum. **TS1′** is then converted to **NT2′**, in which a H---H bond is completely cleaved, and the resulting hydrogermylene moiety is stabilized by the coordination of a newly formed H–Mo bond to the 4p-orbital of Ge. This route thus suggests that the dihydrogen-activation reaction is promoted efficiently on metallogermylenes by the cooperative action of the metal and the ligand. Another route, which is high in energy at ca. 20 kJ/mol, includes **TS2′** which is related to the “oxidative-addition” type of reaction occurring exclusively on the Ge center, proposed for the H_2_ activation by organotetrylenes (Figure 1a).

**Figure 5:**
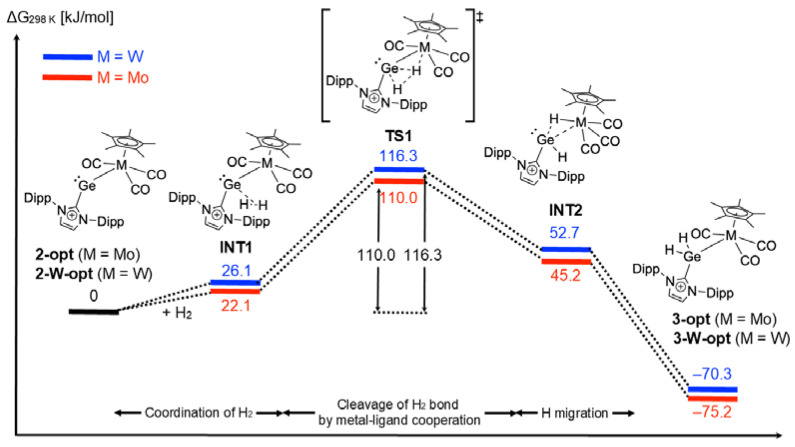
Gibbs free energy profile for the dihydrogen activation using cationic metallogermylenes **2-opt** (M = Mo) and **2-W-opt** (M = W), calculated at the M06/LANL2DZ[Mo,W]:6-31+G(d,p)[H, C, N, O, Ge] (SMD: C_6_H_5_F) level. “‡” indicates a transition state.

Based on these results, we further evaluated the effect of metals (Mo and W) on the activation energy for the lower route using real systems (Figure 5). The activation barrier in the Mo system was calculated to be 110.0 kJ/mol, which is lower than that (116.3 kJ/mol) in the W system, in agreement with the aforementioned experimental results. Orbital analysis suggests significant involvement of d-orbitals in HOMO and LUMO (Figure 3b) as well as TS1 and intermediates in the reaction profiles (Figures S25–S27). The d-orbital component leads to the lowering of their energy levels and activation energy by changing the metal from W to Mo. In this sense, the dihydrogen activation mechanism by metallogermylenes can be differentiated from that promoted by organogermylenes.

## 3. Experimental Section

### 3.1. General Procedures

All manipulations were performed under an argon atmosphere in a glovebox or under a nitrogen atmosphere using Schlenk-line techniques. All solvents were dried and stored under argon over 4 Å molecular sieves in a glovebox before use or directly transferred into the reaction apparatus using Schlenk-line techniques.

### 3.2. Solvents, Reagents, and Precursors

*n*-Hexane was dried using a Glass Contour alumina column (Nikko Hansen & Co., Ltd., Osaka, Japan). Toluene was dried using an MBRAUN Solvent Purification System (München, Germany) and further dried by storage over CaH_2_, followed by distillation. C_6_H_5_F, *o*-DCB-*d*_4_, and THF-*d*_8_ were dried over CaH_2_ and distilled prior to use. GeCl_2_(IPr) [33] and Na[BAr^F^_4_] [34] were synthesized according to literature methods. Li[Cp*Mo(CO)_3_] was synthesized by a literature method [35] with a slight modification (see Section 3.6.1).

### 3.3. Spectroscopic Measurements

NMR spectra were obtained on a Bruker AVANCE III 400 Fourier-transform spectrometer (Billerica, MA, USA) (^1^H: 400.1 MHz; ^11^B: 128.4 MHz; ^13^C: 100.6 MHz; ^19^F: 376.5 MHz). Chemical shifts are reported in parts per million. Coupling constants (*J*) are given in Hz. ^1^H NMR chemical shifts are referenced to residual partially protonated solvents in the fully deuterated solvents (C_6_D_6_: 7.15 ppm; THF-*d*_8_: 3.58 ppm; *o*-DCB-*d*_4_: 6.93 ppm). ^13^C{^1^H} NMR chemical shifts are referenced to the peaks of the deuterated solvents (C_6_D_6_: 128.0 ppm; THF-*d*_8_: 66.2 ppm; *o*-DCB-*d*_4_: 127.2 ppm). ^11^B and ^19^F{^1^H} NMR chemical shifts are referenced to BF_3_·OEt_2_ (0.0 ppm) and C_6_H_5_CF_3_ (63.7 ppm), respectively, as external standards. All NMR data were collected at room temperature unless otherwise indicated. IR spectra were recorded on a HORIBA FT-720 spectrometer at room temperature (Kyoto, Japan), Elemental analyses were performed using a J-Science Lab JM11 microanalyzer (Kyoto, Japan), and high resolution mass spectroscopy (HRMS) was performed using a Bruker Daltonics solarix 9.4T spectrometer (Billerica, MA, USA) operating in the electrospray ionization (ESI) mode at the Research and Analytical Center for Giant Molecules at Tohoku University (Japan).

### 3.4. X-Ray Crystallographic Analysis of ***2*** and ***3***

Single crystals of **2** and **3** were obtained from slow recrystallization in C_6_H_5_F and *n*-hexane at −30 °C in a glovebox filled with argon. Diffraction measurements were recorded on a RIGAKU RAXIS-RAPID Imaging Plate diffractometer (Tokyo, Japan) using graphite-monochromated Mo Kα radiation (λ = 0.71073 A) at 150(2) K. Numerical absorption collections were made using the program NUMABS [36]. The structures of **2** and **3** were solved by Patterson and Fourier-transform methods (SHELXS-97) [37] and refined by the full-matrix least-squares technique on F2 using the SHELXL-2016 [38] and Yadokari-XG 2009 [39,40] software packages. In the crystals of **2** and **3**, two crystallographic independent molecules were found per unit cell, and these are structurally almost identical in each case. The positions of hydrogen atoms on Ge–H in **3** were attributed based on the difference in the Fourier-transform electron-density map and refined isotropically. All other hydrogen atoms were placed at their geometrically calculated positions. For both cases, although there were non-avoidable disordering moieties in one Cp* group and the counter anion, the main structural parameters were well reproduced by DFT calculations. CCDC-2401509 (**2**) and CCDC-2401510 (**3**) contain supplementary crystallographic data for this paper. These data can be obtained free of charge from The Cambridge Crystallographic Data Centre via www.ccdc.cam.ac.uk/data_request/cif (accessed on 9 November 2024).

### 3.5. Computation Details

Theoretical calculations were performed using real (**2-opt** and **3-opt**) and model molecules (**2′-opt** and **3′-opt**) (Chart S1). For the model molecules (**2′-opt** and **3′-opt**), the Cp* group was changed to a Cp group, and the Dipp group was replaced by a slightly smaller Ph group. Geometries were optimized using the M06 functional in the gas phase [41]. For Mo and W, the LANL2DZ [42] basis sets were employed, whereby the core electrons were replaced with the effective core potentials (ECPs). For the other elements, the 6-31G(d,p) [43] basis sets were employed. For the simulation of the absorption spectrum of **2**, TD-DFT calculations were carried out on the optimized geometry at the M06/LANL2DZ[Mo]:6-31+G(d,p)[H, C, Ge, N, O] level. For each stationary structure, the vibrational frequencies were calculated at the same computational level in order to determine whether this structure is an equilibrium structure or a transition state. Furthermore, the intrinsic reaction coordinate (IRC) was determined, which clearly showed the imaginary frequency along the reaction coordinate, in order to ensure that the transition states connect to the corresponding reactants and products. In these calculations, the solvent effects of C_6_H_5_F were considered by using a solvent model based on density [44]. The Gibbs free energy was evaluated under 298.15 K and 1 atm experimental conditions. All calculations were carried out using the Gaussian16 [45] program package.

### 3.6. Synthesis of the Molybdenum Complexes (Figures S1–S18)

#### 3.6.1. Modified Synthesis of [Cp*Mo(CO)_3_][Li(thf)_1.5_]

[Cp*Mo(CO)_3_][Li(thf)_1.5_] was synthesized slightly modifying the literature procedure [34] in the purification process that is reported here in detail. Pentamethylcyclopentadiene (2.76 g, 20.3 mmol) dissolved in THF (30 mL) at room temperature was treated dropwise with *n*-BuLi (1.5 M, 15.0 mL, 22.5 mmol). Then, after stirring for 1 h, hexacarbonylmolybdenum (5.35 g, 20.3 mmol) was added. After stirring under reflux conditions for 24 h and subsequent cooling to room temperature, the solvent was removed under reduced pressure to afford a reddish-brown viscous solid (crude yield: 7.80 g). The obtained solid was treated with THF (~15 mL) and centrifuged, and the supernatant was decanted, before all volatiles were removed from the supernatant to afford [Cp*Mo(CO)_3_][Li(thf)_1.5_] as a reddish-brown solid (4.63 g, 10.8 mmol, 53% yield). ^1^H NMR (400 MHz, THF-*d*_8_, *δ*) 2.01 (s, 15H, Cp*); ^7^Li NMR (156 MHz, THF-*d*_8_, *δ*) –0.94; ^13^C{^1^H} NMR (101 MHz, THF-*d*_8_, *δ*) 11.0 (C_5_Me_5_), 99.3 (C_5_Me_5_), 238.9 (CO).

#### 3.6.2. Synthesis of Cp*Mo(CO)_3_GeCl(IPr) (**1**)

GeCl_2_(IPr) (102.7 mg, 0.193 mmol) was added to [Cp*Mo(CO)_3_][Li(thf)_1.5_] (88.1 mg, 0.205 mmol) in toluene (3 mL). After stirring for 30 min at room temperature, the mixture was filtered and the filtrate was dried. The obtained yellow solid was washed with hexane (3 × ~1 mL) and dried under reduced pressure to afford Cp*Mo(CO)_3_GeCl(IPr) (**1**) as a yellow powder (142.3 mg, 0.176 mmol, 91% yield). ^1^H NMR (400 MHz, C_6_D_6_, *δ*) 0.98 (d, ^3^*J*_HH_ = 6.8 Hz, 6H, CH(C*H*_3_)_2_), 0.99 (d, ^3^*J*_HH_ = 6.8 Hz, 6H, CH(C*H*_3_)_2_), 1.54 (d, ^3^*J*_HH_ = 6.8 Hz, 6H, CH(C*H*_3_)_2_), 1.64 (s, 15H, Cp*), 1.66 (d, ^3^*J*_HH_ = 6.8 Hz, 6H, CH(C*H*_3_)_2_), 3.07 (sept, ^3^*J*_HH_ = 6.8 Hz, 2H, C*H*(CH_3_)_2_), 3.18 (sept, ^3^*J*_HH_ = 6.8 Hz, 2H, C*H*(CH_3_)_2_), 6.56 (s, 2H, NCH), 7.17–7.30 (m, 6H, Ar-H); ^13^C{^1^H} NMR (101 MHz, C_6_D_6_, *δ*) 10.1 (C_5_*Me*_5_), 23.01 (CH(*C*H_3_)_2_), 23.08 (CH(*C*H_3_)_2_), 26.3 (CH(*C*H_3_)_2_), 26.8 (CH(*C*H_3_)_2_), 28.9 (CH(*C*H_3_)_2_), 29.1 (*C*H(CH_3_)_2_), 103.1 (*C*_5_Me_5_), 124.2 (*m*-C, Dipp), 124.5 (*m*-C, Dipp), 125.2 (NCCN), 131.0 (*p*-C, Dipp), 134.7 (*ipso*-C, Dipp), 146.17 (*o*-C, Dipp), 146.20 (*o*-C, Dipp), 179.2 (NCN), 226.7 (CO), 230.5 (CO), 238.7 (CO); IR (KBr, cm^−1^) 1846 (s, CO), 1884 (s, CO), 1953 (s, CO).

#### 3.6.3. Synthesis of [Cp*Mo(CO)_3_Ge(IPr)][BAr^F^_4_] (**2**)

(a) NMR experiment: Na[BAr^F^_4_] (10.2 mg, 11.5 μmol) was added to a fluorobenzene solution (0.4 mL) of **1** (10.0 mg, 12.3 μmol) in an NMR tube with a Teflon valve, to which a sealed capillary tube containing a solution of Cp_2_Fe (an external standard) in C_6_D_6_ (for a lock solvent) was inserted. The reaction was monitored by ^1^H NMR spectrum, which showed nearly quantitative formation of [Cp*Mo(CO)_3_Ge(IPr)][BAr^F^_4_] (**2**) soon after the addition.

(b) Preparative-scale experiment: In a 50 mL Schlenk flask, Na[BAr^F^_4_] (106.6 mg, 120 μmol) was added to **1** (98.3 mg, 121 μmol) in fluorobenzene (5.0 mL). The resulting mixture was stirred at room temperature to yield **2** as air- and moisture-sensitive yellow-green crystals (83.7 mg, 51.0 μmol, 43% yield). ^1^H NMR (400 MHz, C_6_H_5_F, *δ*) 1.03 (d, ^3^*J*_HH_ = 6.8 Hz, 12H, CH(C*H*_3_)_2_), 1.13 (d, ^3^*J*_HH_ = 6.8 Hz, 12H, CH(C*H_3_*)_2_), 1.55 (s, 15H, Cp*), 2.82 (sept, ^3^*J*_HH_ = 6.8 Hz, 4H, C*H*(CH_3_)_2_); ^1^H NMR (400 MHz, *o*-DCB-*d*_4_, *δ*) 1.07 (d, ^3^*J*_HH_ = 6.8 Hz, 12H, CH(C*H*_3_)_2_), 1.14 (d, ^3^*J*_HH_ = 6.8 Hz, 12H, CH(C*H*_3_)_2_), 1.62 (s, 15H, Cp*), 2.80 (sept, ^3^*J*_HH_ = 6.8 Hz, 4H, C*H*(CH_3_)_2_), 7.10 (d, 2H, ^3^*J*_HH_ = 7.8 Hz, *m*-H, Dipp), 7.31 (t, 2H, ^3^*J*_HH_ = 7.8 Hz, *p*-H, Dipp), 7.59 (br s, 4H, *p*-H, ArF), 7.73 (s, 2H, NCH), 8.17 (br s, 8H, *o*-H, Ar^F^); ^13^C{^1^H} NMR (101 MHz, *o*-DCB-*d*_4_, δ) 10.1 (C_5_*Me*_5_), 108.5 (*C*_5_Me_5_), 124.8 (q, ^1^*J*_CF_ = 272 Hz, CF_3_), 128.4 (NCH), 129.4 (m, *m*-C, Ar^F^), 135.1 (*o*-C, Ar^F^), 145.3 (*o*-C, Dipp), 162.3 (q, ^4^*J*_CF_ = 50 Hz, *ipso*-C, ArF), 188.5 (NCN), 223.0 (CO); ^11^B NMR (128 MHz, *o*-DCB-*d*_4_, *δ*) –6.26 (BAr^F^_4_); ^19^F{^1^H} NMR (376 MHz, *o*-DCB-*d*_4_, δ) –62.1 (s, CF_3_); IR (KBr, cm^−1^) 1942 (s, CO), 1961 (s, CO), 2029 (s, CO); UV–vis (C_6_H_5_F, 1.5 × 10^−2^ M, *λ*_max_ nm) 692 (*ε* = 3.4 × 10^2^ M^−1^ cm^−1^), should. 460 (*ε* = 7.3 × 10^2^ M^−1^ cm^−1^), 383 (*ε* = 1.6 × 10^3^ M^−1^ cm^−1^). Anal. Calcd. for C_72_H_63_BF_24_GeN_2_O_3_Mo: C, 52.74; H, 3.87; N, 1.71. Found: C, 52.36; H, 3.99; N, 1.75.

#### 3.6.4. Reaction of **2** with Dihydrogen: Formation of [Cp*(CO)_3_MoGeH_2_(IPr)][BAr^F^_4_] (**3**)

Complex **2** was extremely moisture sensitive and was prepared in situ from **1** (14.4 mg, 17.8 μmol) and Na[BAr^F^_4_] (13.8 mg, 15.6 μmol) in fluorobenzene (0.4 mL) in an NMR tube with a Teflon valve. A sealed capillary tube containing a solution of Cp_2_Fe (an external standard) in C_6_D_6_ (for a lock solvent) was inserted into the NMR tube. The resulting mixture was degassed, and H_2_ (1 atm) was introduced. The reaction mixture was heated at 60 ºC and was monitored by ^1^H NMR, which showed nearly quantitative formation of [Cp*(CO)_3_MoGeH_2_(IPr)][BAr^F^_4_] (**3**) after 5 h. During the reaction, the color of the mixture gradually changed from green to yellow. Complete isolation of **3** was hampered by concomitant non-separable [IPrH][BAr^F^_4_] and gradual decomposition of **3** during the recrystallization. Nevertheless, the structure of **3** was elucidated by the following data and 2D NMR, SC-XRD, and DFT study. ^1^H NMR (400 MHz, C_6_H_5_F, *δ*) 1.00 (d, ^3^*J*_HH_ = 6.8 Hz, 12H, CH(C*H*_3_)_2_), 1.31 (d, ^3^*J*_HH_ = 6.8 Hz, 12H, CH(C*H*_3_)_2_), 1.42 (s, 15H, Cp*), 2.47 (sept, ^3^*J*_HH_ = 6.8 Hz, 4H, CH(C*H*_3_)_2_) 3.94 (s, 2H, GeH_2_). ^13^C{^1^H} NMR (101 MHz, C_6_H_5_F, *δ*) 9.4 (C_5_*Me*_5_), 21.9, 25.6 (CH(*C*H_3_)_2_), 29.1 (*C*H(CH_3_)_2_), 104.6 (*C*_5_Me_5_), 117.8 (*p*-C, Ar^F^), 125.1 (*m*-C, Dipp), 135.2 (*o*-C, ArF), 145.3 (*o*-C, Dipp), 160.2 (NCN), 162.6 (q, ^4^*J*_CF_ = 50 Hz, *ipso*-C, Ar^F^), 226.9 (CO), 231.5 (CO); ^11^B NMR (128 MHz, C_6_H_5_F, *δ*) –6.16 (BAr^F^_4_); ^19^F{^1^H} NMR (376 MHz, C_6_H_5_F, *δ*) –62.6 (s, CF_3_); IR (KBr, cm^−1^) 1930 (s, CO), 1955 (s, CO), 2015 (s, CO); HRMS (ESI, positive): *m*/*z* calcd. for [^12^C_40_^1^H_53_^74^Ge^98^Mo^14^N_2_^16^O_3_]^+^ ([Cp*(CO)_3_MoGeH_2_(IPr)]^+^): 781.2317, found: 781.2315.

## 4. Conclusions

In this study, we have synthesized and characterized neutral and cationic metallogermylenes of molybdenum. The cationic molybdogermylene reacted with dihydrogen much faster than the tungsten analog to produce a dihydrogermyl complex via H–H bond activation. The observed metal effect on the dihydrogen activation by metallogermylenes was investigated theoretically. Thus, we discovered a lower new reaction pathway that is promoted by metal–ligand cooperative activation between the metal and the divalent germanium moiety of the metallogermylene. This is in sharp contrast to the “oxidative addition” type of activation that occurs on the single element center of usual tetrylenes. The origin of the metal effect can be attributed to the non-negligible contribution of metal d-orbitals in the frontier orbitals of metallogermylenes, which lowers their energy levels by changing the metal from tungsten to molybdenum. These results could provide useful information for the future design of reactive complexes and exploring new inert bond activation systems.

## Data Availability

All data are available in the main text or the Supplementary Materials.

## Supplementary Materials

The following supporting information can be downloaded at https://www.mdpi.com/article/10.3390/molecules29245974/s1, NMR, IR, and UV–vis spectra, X-ray crystallographic data of complexes, and DFT calculations.
