# Supplementary material for: Metal–Ligand Cooperation in Dihydrogen Activation by a Cationic Metallogermylene: Enhanced Activity from Tungsten to Molybdenum
Source: Molecules. 2024 Dec 18;29(24):5974. doi: 10.3390/molecules29245974 (PMC11676882; doi:10.3390/molecules29245974)
Supplement: Supplementary file 1 [file molecules-29-05974-s001.zip › MoGe_Mol_ESI_2_P1-20.pdf]

Communication

Metal–Ligand Cooperation in Dihydrogen Activation by a Cationic Metallogermylene: Enhanced Activity from Tungsten to Molybdenum

Rikiya Matsumoto, Koichi Nagata, Ryo Nakamura, Takahito Watanabe and Hisako Hashimoto *

Department of Chemistry, Graduate School of Science, Tohoku University, 6-3 Aramaki, Aoba-ku, Sendai 980-8578, Japan; cherry.s5085@gmail.com (R.M.); koichi.nagata.d3@tohoku.ac.jp (K.N.); ryo.nakamura.t8@dc.tohoku.ac.jp (R.N.); takahito.watanabe@gmail.com (T.W.)

* Correspondence: hisako.hashimoto.b7@tohoku.ac.jp; Tel.: +81-22-795-6539

Contents

1. Spectra of [Cp*(CO)$_3$Mo][Li(thf)$_{1.5}$] and complexes 1-3 S2

^{1}H, ^{13}C{^{1}H}, and ^{7}Li NMR spectra of [Cp*(CO)$_3$Mo][Li(thf)$_{1.5}$]

^{1}H, ^{13}C{^{1}H} NMR, and IR spectra of 1

^{1}H, ^{13}C{^{1}H}, ^{11}B{^{1}H}, ^{19}F{^{1}H} NMR, and IR spectra of 2

^{1}H, ^{13}C{^{1}H}, ^{11}B{^{1}H}, ^{19}F{^{1}H}, ^{1}H-^{13}C{^{1}H} NMR, and IR spectra of 3

2. X-ray crystallographic data and structures of 2 and 3 S13

3. Theoretical calculations on 2 and 3 S16

DFT optimized structures and selected parameters

Molecular orbital analyses

Gibbs energy profile for the dihydrogen activation by 2

４. Atomic coordinates for the computed complexes and transition states S21

1. Spectra of [Cp*(CO)$_3$Mo][Li(thf)$_{1.5}$] and complexes 1-3

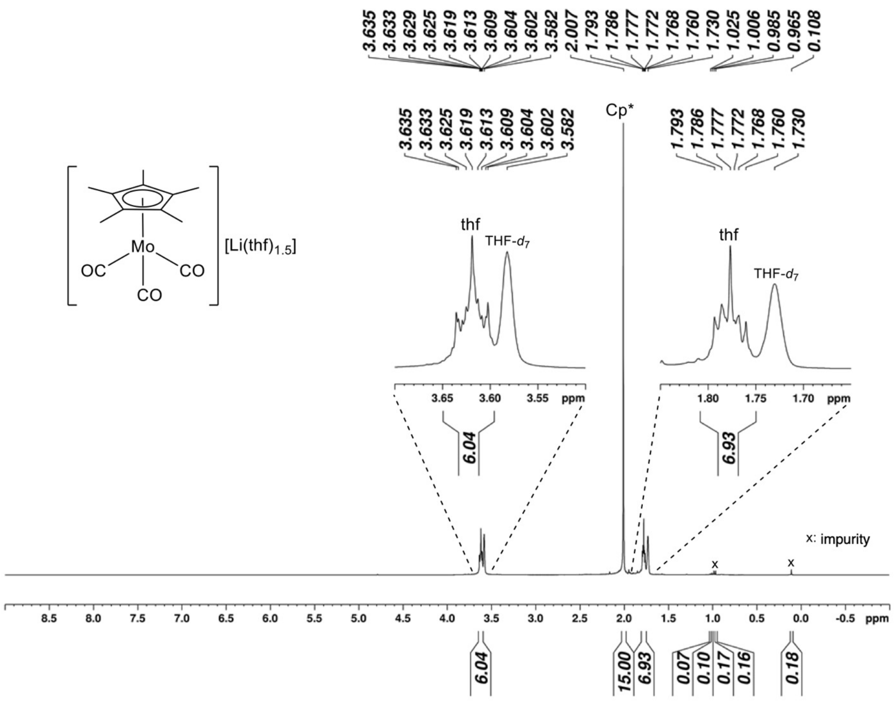

Figure S1. *^{1}H NMR spectrum of [Cp*(CO)$_3$Mo][Li(thf)$_{1.5}$] (400 MHz, THF-d$_8$, 300 K).*

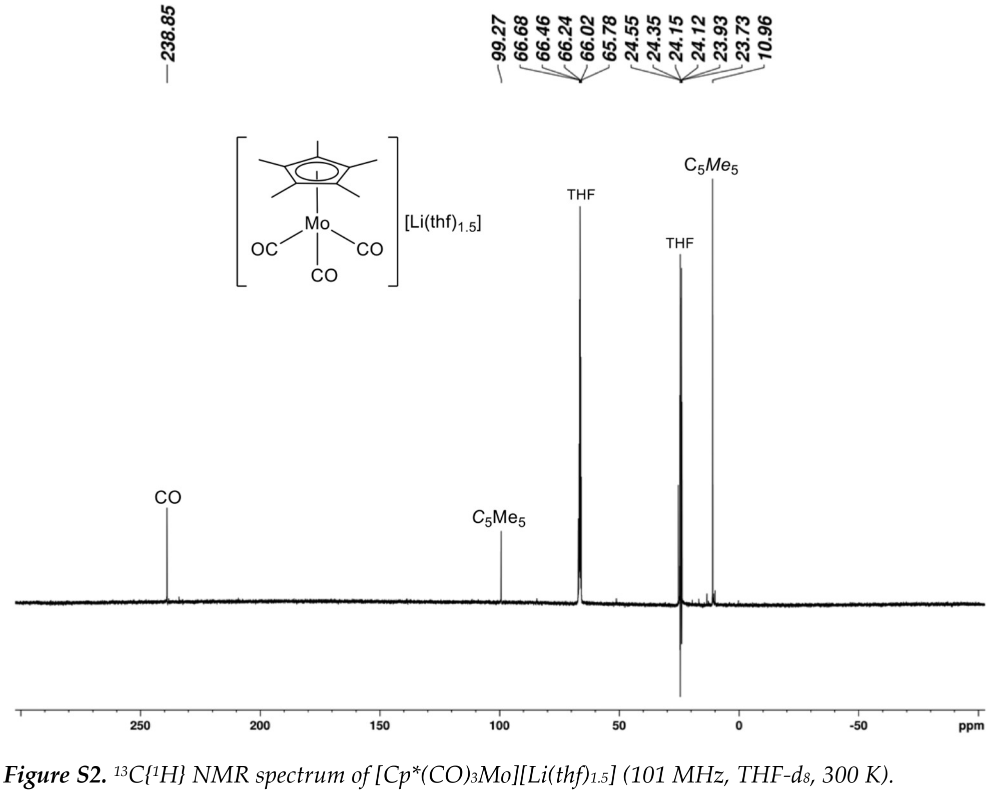

Figure S2. *^{13}C{^{1}H} NMR spectrum of [Cp*(CO)$_3$Mo][Li(thf)$_{1.5}$] (101 MHz, THF-d$_8$, 300 K).*

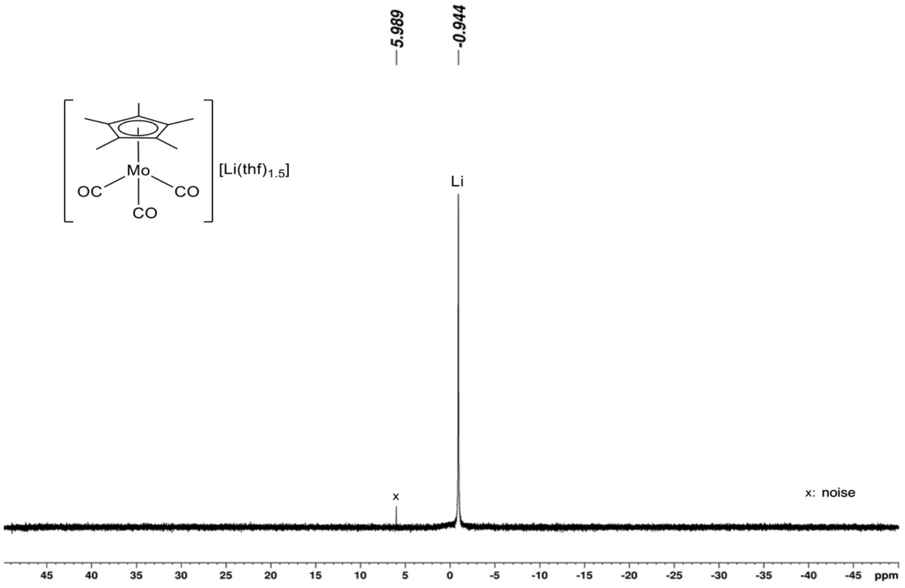

Figure S3. *^{7}Li NMR spectrum of [Cp*(CO)$_3$Mo][Li(thf)$_{1.5}$] (156 MHz, THF-d$_8$, 300 K).*

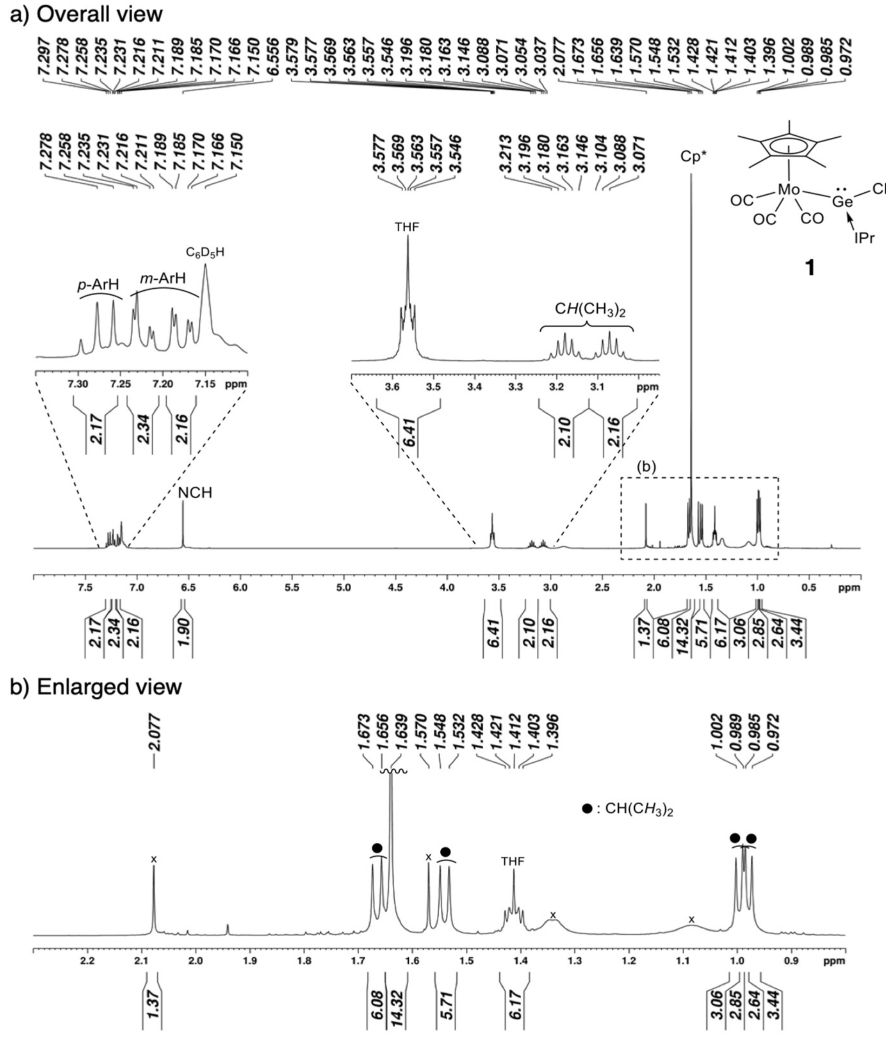

Figure S4. *^{1}H NMR spectrum of **1** (400 MHz, C_6D_6, 300 K). a) Overall view, b) Enlarged view (0.8~2.3 ppm).*

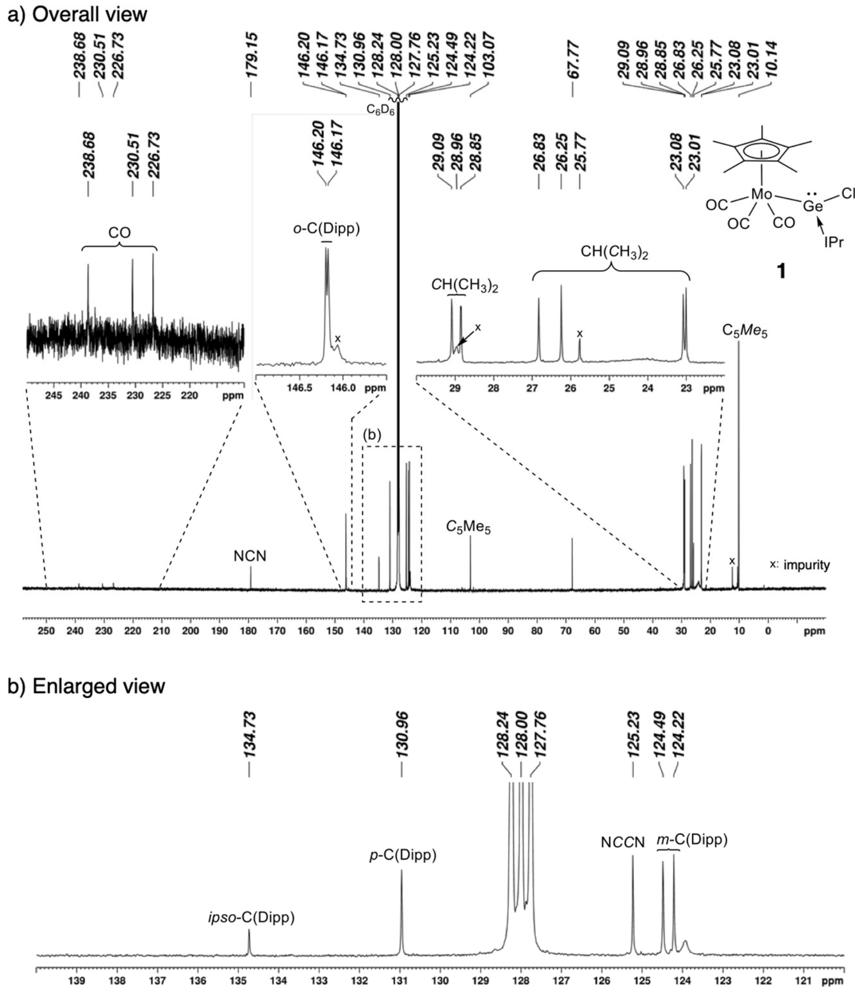

Figure S5. $^{13}C\{^{1}H\}$ *NMR spectrum of* ***1*** *(101 MHz,* C_6D_6*, 300 K). a) Overall view, b) Enlarged view (120~140 ppm)*

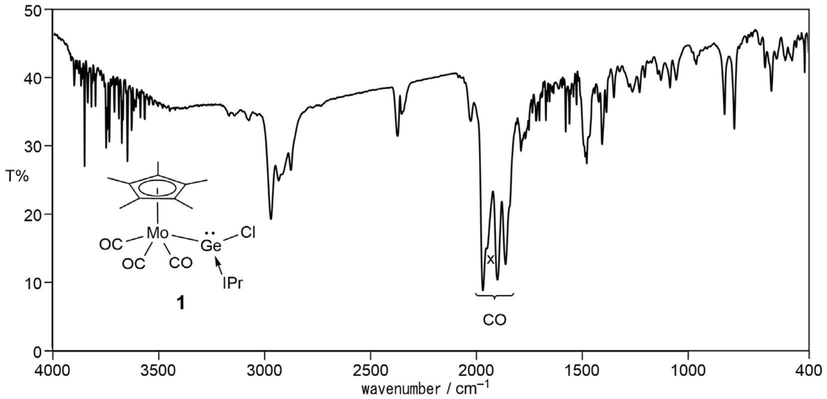

complex	**1**
ν_{CO} / cm^{-1}	1846, 1884, 1953

Figure S6. *FT-IR spectrum of* ***1****.*

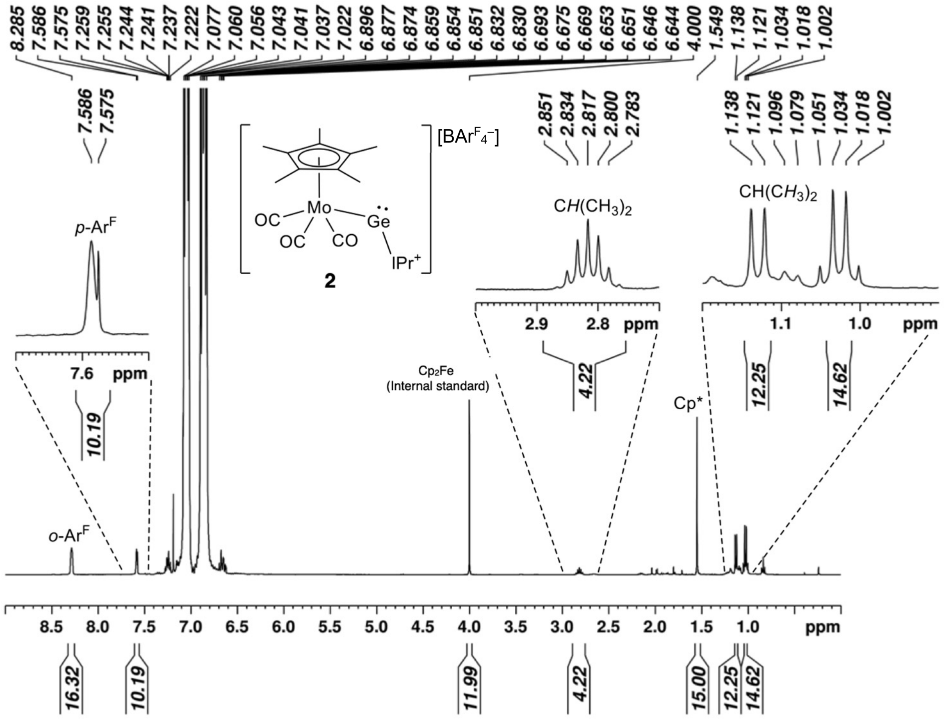

Figure S7. *^{1}H NMR spectrum of* ***2*** *(400 MHz, C_6H_5F + C_6D_6 in a glass capillary, 300 K).*

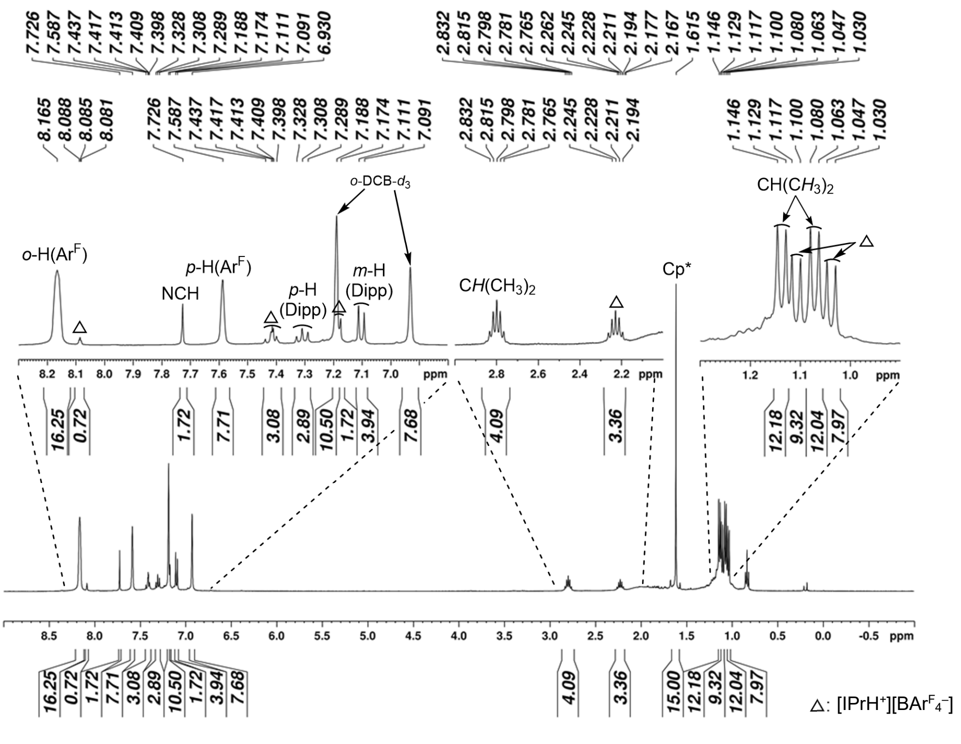

Figure S8. *^{1}H NMR spectrum of* ***2*** *(400 MHz, o-DCB-d_4, 300 K).*

a) Overall view

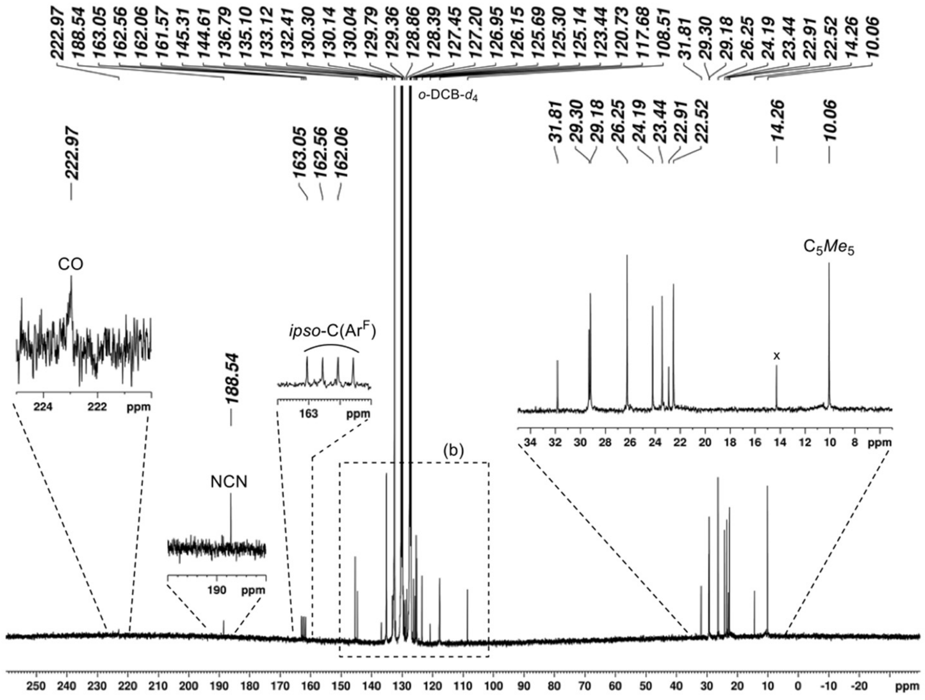

b) Enlarged view

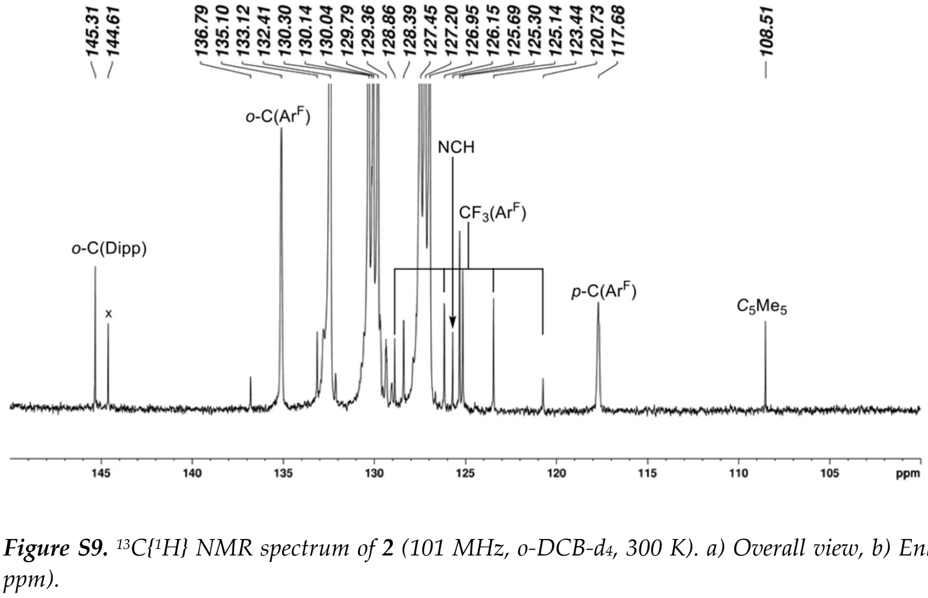

Figure S9. *$^{13}C\{^1H\}$ NMR spectrum of **2** (101 MHz, o-DCB-d_4, 300 K). a) Overall view, b) Enlarged view (100~150 ppm).*

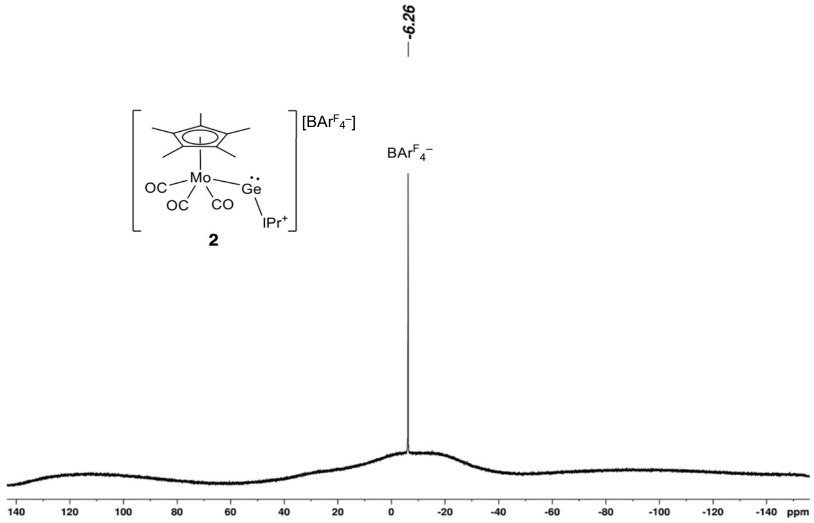

Figure S10. *$^{11}B\{^{1}H\}$ NMR spectrum of* ***2*** *(128 MHz, o-DCB-d$_4$, 300 K).*

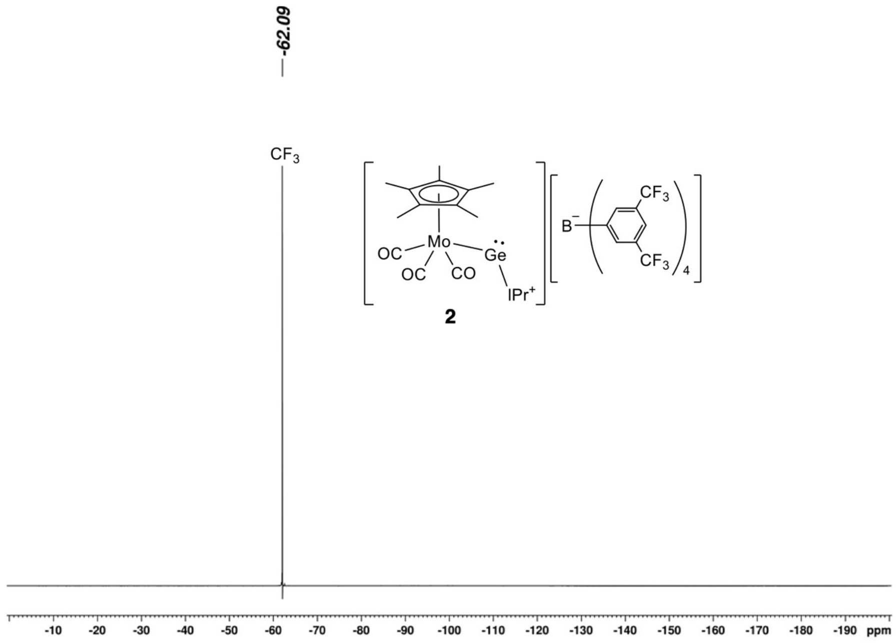

Figure S11. *$^{19}F\{^{1}H\}$ NMR spectrum of* ***2*** *(376 MHz, o-DCB-d$_4$, 300 K).*

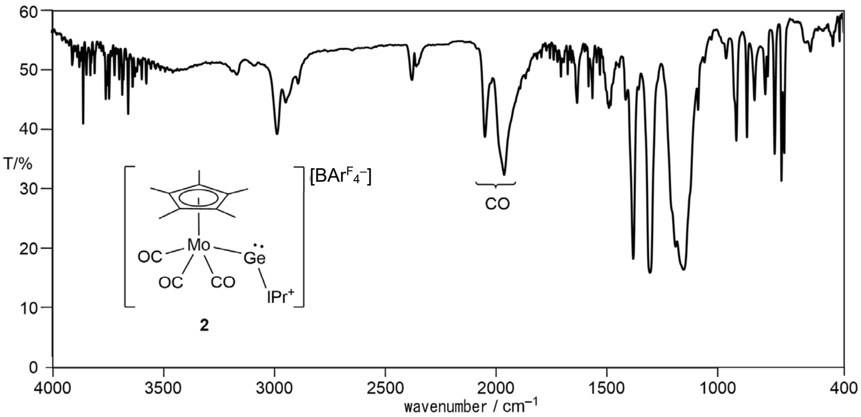

Complex	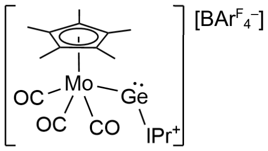<br>**2**
ν_{CO} / cm^{-1} (KBr)	1942, 1961, 2029
ν_{CO} / cm^{-1} (DFT)	2002, 2038, 2096

Figure S12. *FT-IR spectrum of* **2** *and comparison of observed νCO bands with calculated ones.*

(a) Overall view

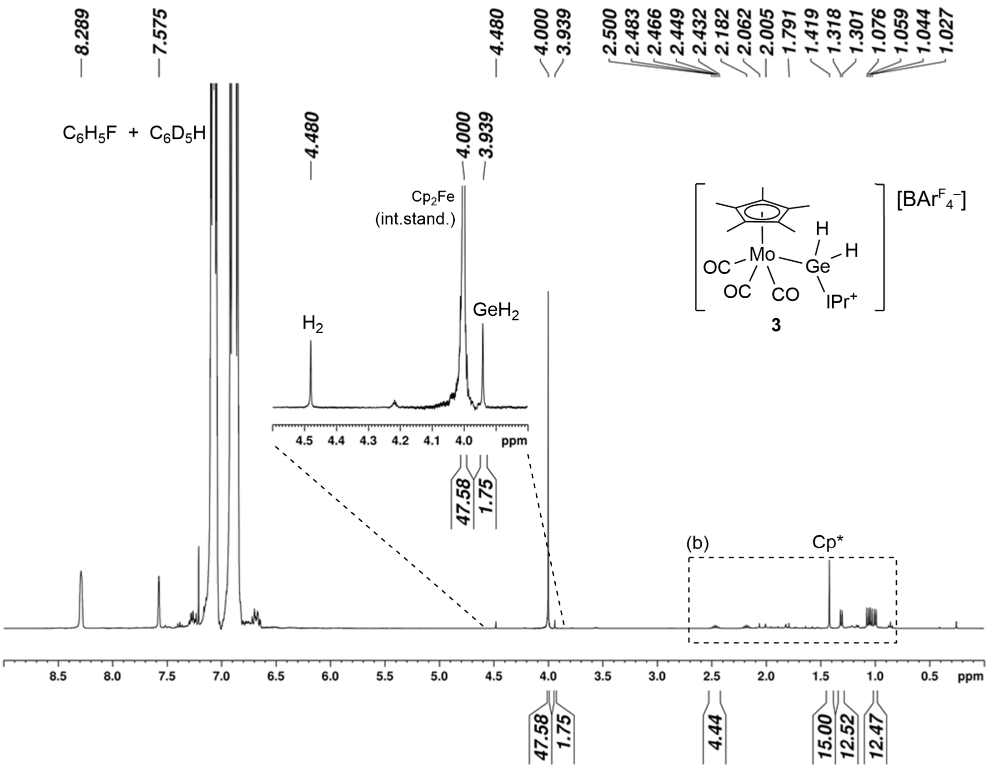

(b) Expanded view

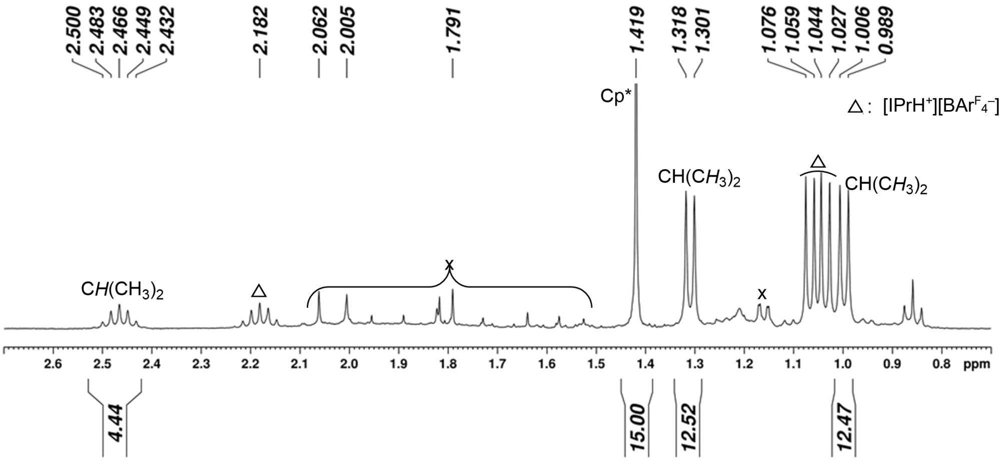

Figure S13. *^{1}H NMR spectrum obtained after the reaction of* ***1*** *with H_2 (400 MHz, C_6H_5F + C_6D_6 in a glass capillary, 300 K).*

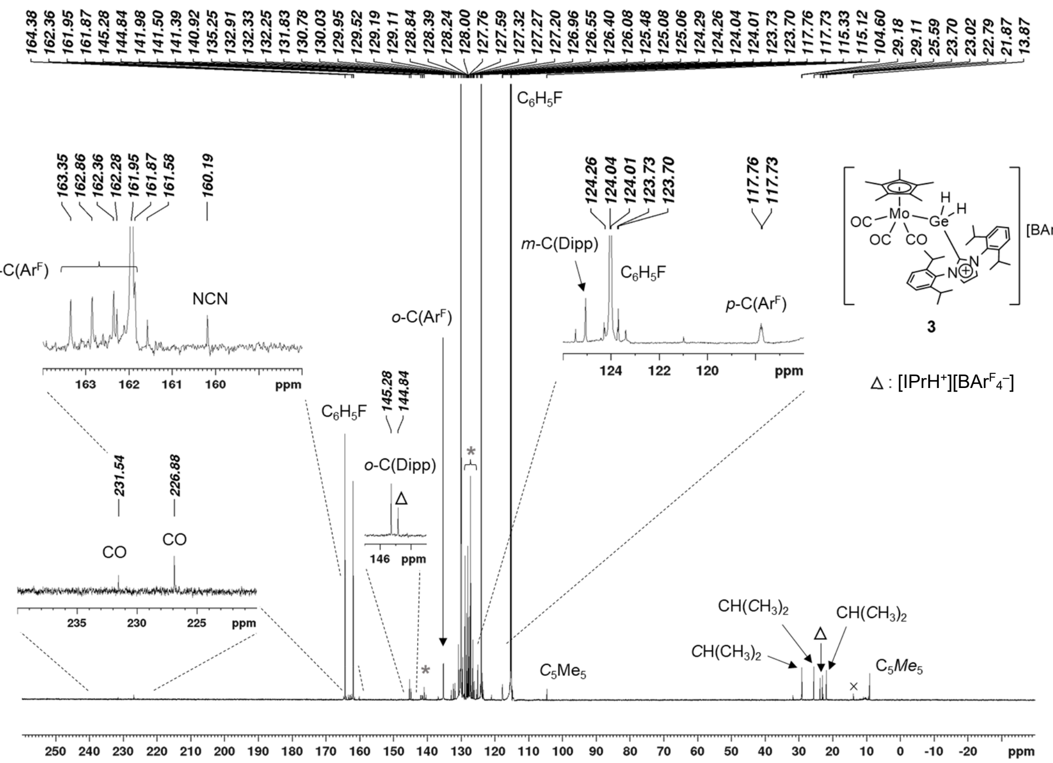

Figure S14. *$^{13}C\{^1H\}$ NMR spectrum of [Cp*(CO)$_3$MoGeH$_2$(IPr)](BAr$^F_4$)* ***(3)*** *(101 MHz, C_6H_5F + C_6D_6 in a glass capillary, 300 K)*

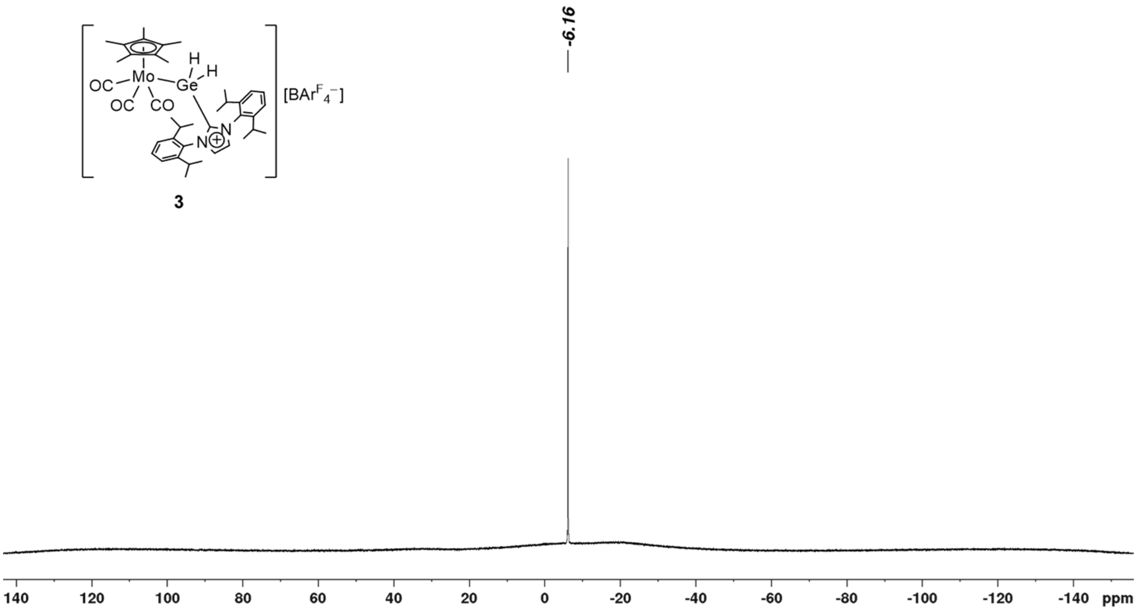

Figure S15. *^{11}B NMR spectrum of [Cp*(CO)$_3$MoGeH$_2$(IPr)](BAr$^F_4$)* ***(3)*** *(128 MHz, C_6H_5F + C_6D_6 in a glass capillary, 300 K).*

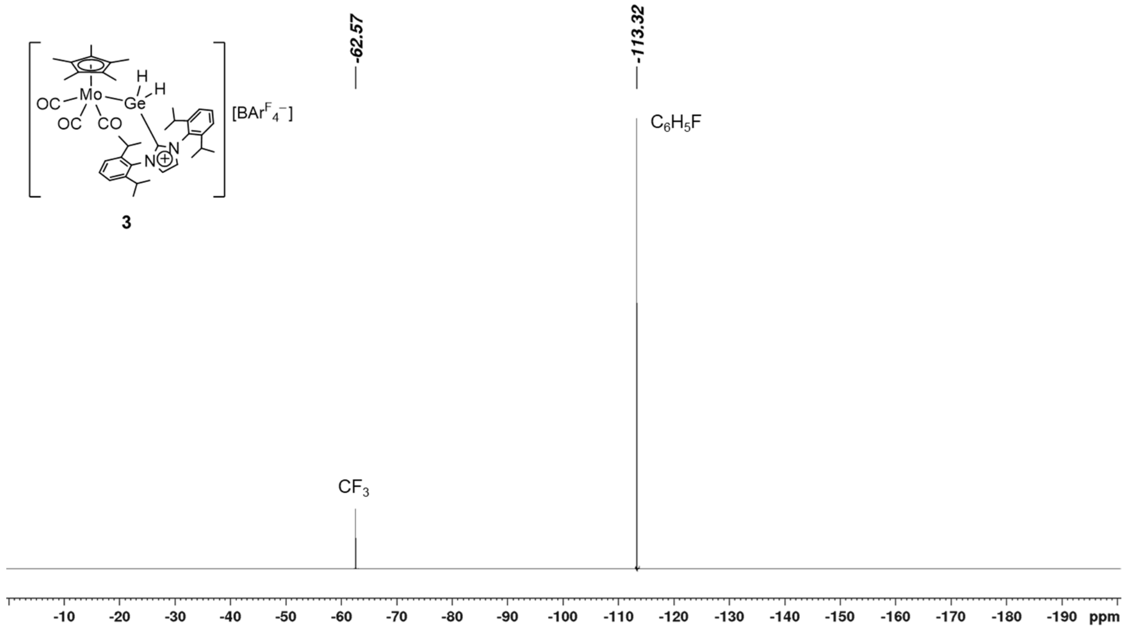

Figure S16. *$^{19}F\{^1H\}$ NMR spectrum of [Cp*(CO)$_3$MoGeH$_2$(IPr)](BAr^F$_4$) (**3**) (376 MHz, C_6H_5F + C_6D_6 in a glass capillary, 300 K).*

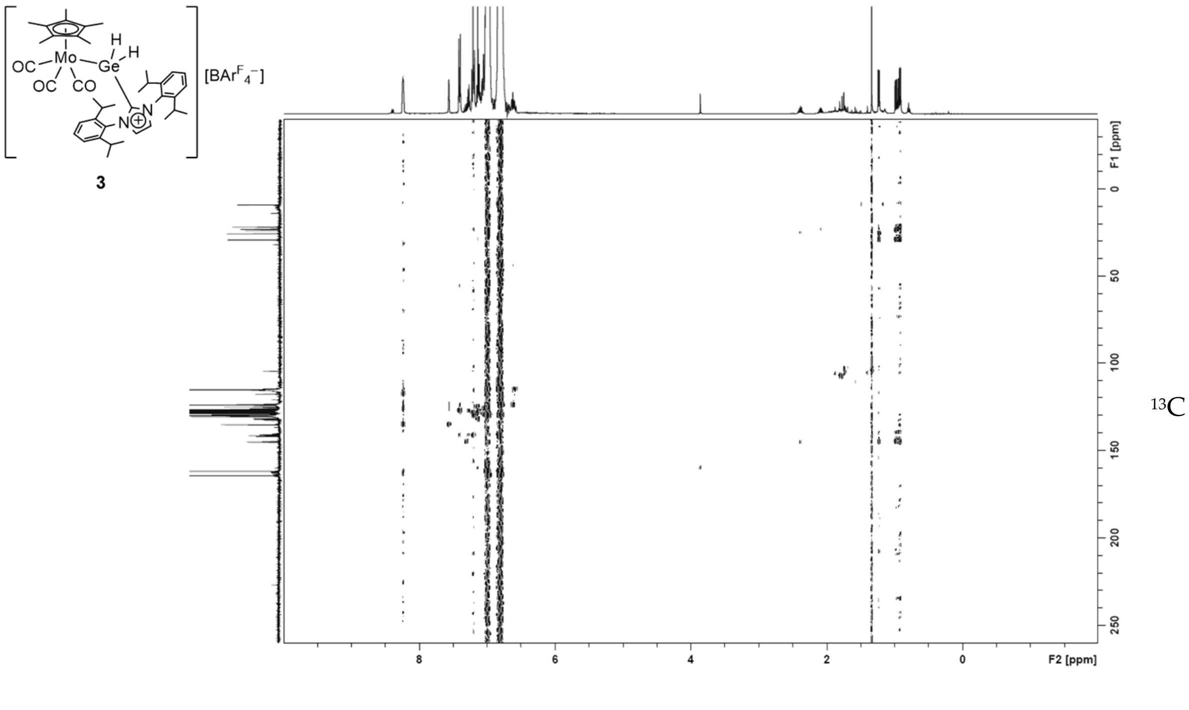

Figure S17-1. ***^1H -^{13}C**$\{^1H\}$ HMBC NMR spectrum of **3** (400 MHz, C_6H_5F + C_6D_6 in a glass capillary, 300 K).*

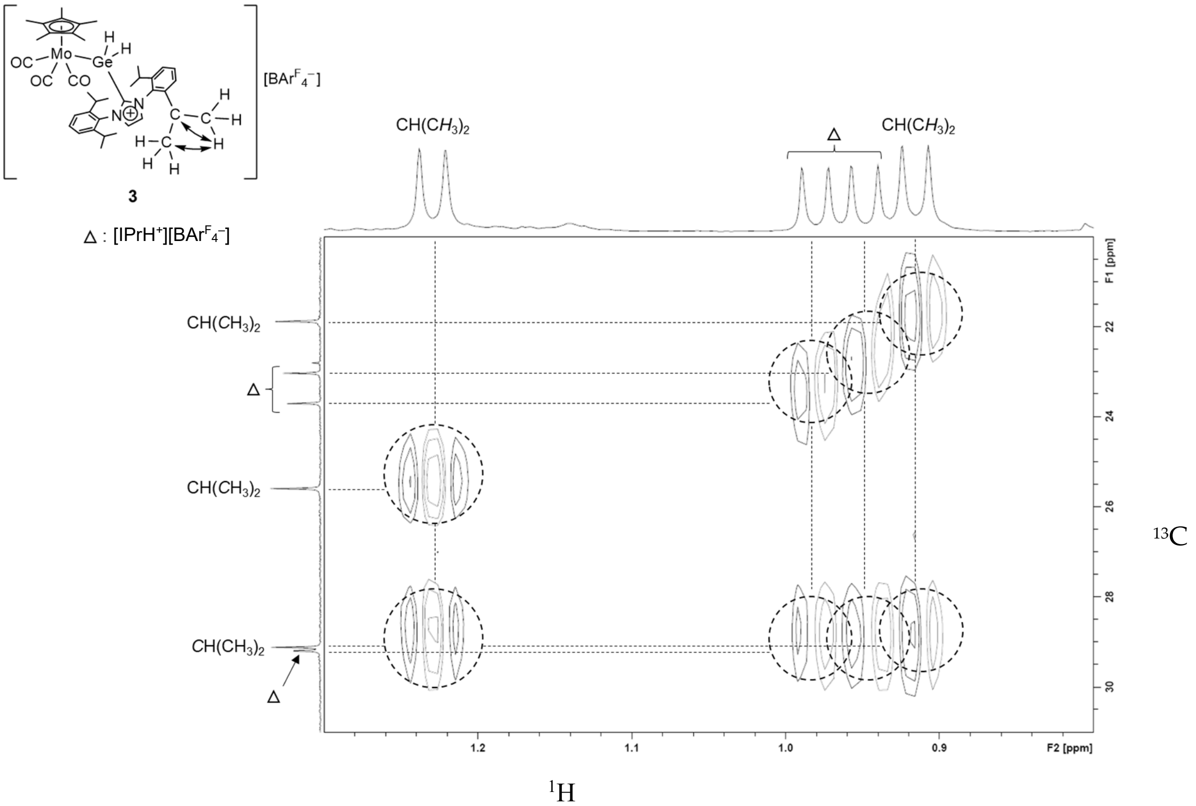

Figure S17-2. *Enlarged* ***^{1}H -^{13}C***{^{1}H} *HMBC NMR spectrum of* ***3*** *(400 MHz, C_6H_5F + C_6D_6 in a glass capillary, 300 K). [C_6H_5F, 300 K; range 0.8–1.3 ppm (^{1}H), 20–31 ppm (^{13}C)]*

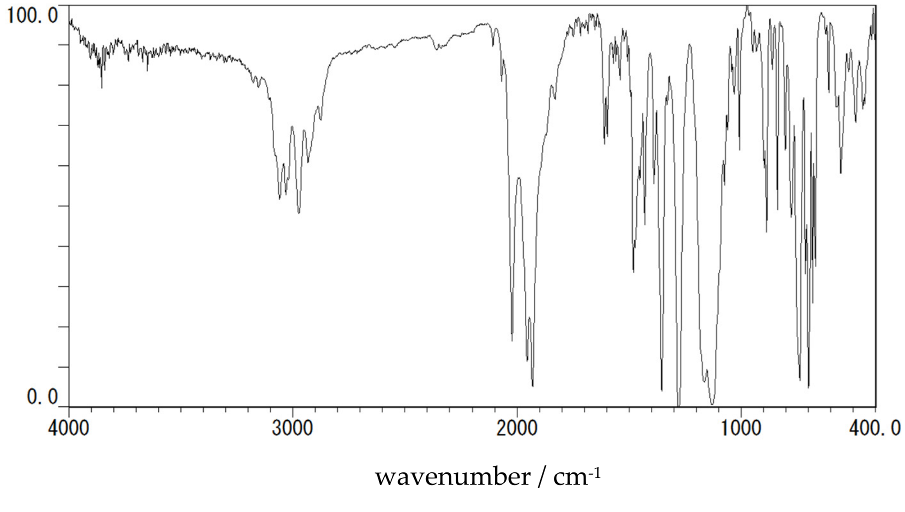

Complex	**3**
ν_{CO} / cm^{-1} (KBr)	1930, 1955, 2021
ν_{CO} / cm^{-1} (DFT)	1926, 1958, 2014

Figure S18. *IR spectrum of [Cp*(CO)$_3$MoGeH$_2$(IPr)](BAr$^{F}_4$)* ***(3)*** *(KBr) and comparison of observed vCO bands with calculated ones.*

2. X-ray crystallographic data and structures of 2 and 3

Table S1. Crystal Data for [Cp*(OC)$_3$MoGe(IPr)][BAr^F$_4$] (**2**) and [Cp*(OC)$_3$MoGeH$_2$(IPr)][BAr^F$_4$] (**3**).

	**2**	**3**
Formula	$C_{72}H_{63}MoGeN_2O_3BF_{24}$	$C_{72}H_{65}MoGeN_2O_3BF_{24}$
Formula weight	1639.58	1641.60
Crystal dimensions/mm^3	0.630 × 0.540 × 0.530	0.380 × 0.330 × 0.110
Temperature/K	150.15	150.15
Crystal system	triclinic	triclinic
Space group	*P*-1 (#2)	*P*-1 (#2)
Lattice parameters		
a/Å	14.9607 (3)	14.9039(5)
b/Å	18.6974(4)	18.6076(6)
c/Å	26.8650(5)	27.7277(8)
α/deg	97.4260(10)	96.5310(10)
β/deg	91.8860(10)	92.1340(10)
γ/deg	101.6260(10)	102.0430(10)
V/Å^3	7285.5(3)	7456.8(4)
Z	4	4
D_{calcd}/g•cm^{-3}	1.495	1.462
μ/mm^{-1}	0.693	0.677
θ/deg	1.268 to 27.484	1.270 to 27.484
No. of reflections	104954	90168
Independent reflections	33114	33264
R_{int}	0.0685	0.0830
Completeness to θ/%	99.6	98.5
R_1 [$I > 2\sigma(I)$]	0.0710	0.0749
wR_2 (all data)	0.1704	0.1734
Largest diff. peak/e•Å^{-3}	2.286	1.552
Largest diff. hole/e•Å^{-3}	–1.646	–0.898
Goodness-of-fit on F^2	1.041	1.046

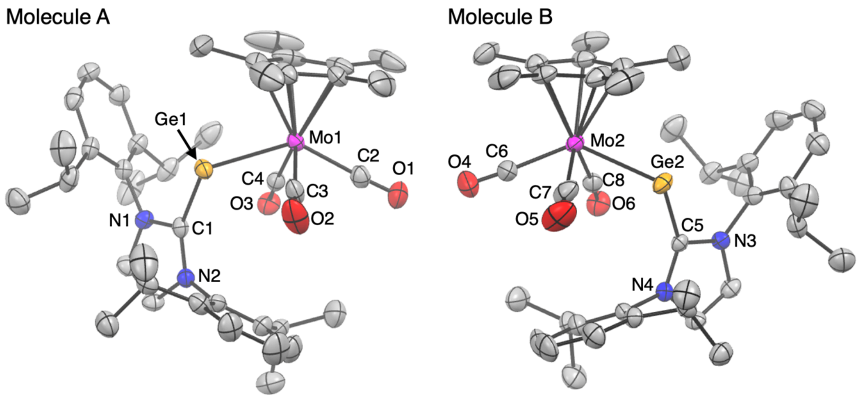

Figure S19. *Molecular structure of* ***2*** *in the crystalline state with thermal ellipsoids at 50% probability. All hydrogen atoms and [B(C_6F_5)$_4$] counter anions are omitted for clarity.*

Table S2. Selected bond lengths [Å] and bond angles [°] in **2**.

Molecule A		Molecule B	
Bond lengths [Å]			
Mo(1)–Ge(1)	2.5701(6)	Mo(2)–Ge(2)	2.5713(6)
Ge(1)–C(1)	2.048(4)	Ge(2)–C(5)	2.041(4)
Mo(1)–C(2)	1.977(5)	Mo(2)–C(6)	1.987(5)
Mo(1)–C(3)	1.994(5)	Mo(2)–C(7)	1.995(5)
Mo(1)–C(4)	1.987(5)	Mo(2)–C(8)	1.976(5)
C(2)–O(1)	1.147(6)	C(6)–O(4)	1.151(5)
C(3)–O(2)	1.139(5)	C(7)–O(5)	1.137(6)
C(4)–O(3)	1.151(5)	C(8)–O(6)	1.147(6)
Bond angles [°]			
Mo(1)–Ge(1)–C(1)	112.45(11)	Mo(2)–Ge(2)–C(5)	112.88(11)
C(2)–Mo(1)–Ge(1)	70.77(13)	C(6)–Mo(2)–Ge(2)	72.5(2)
C(3)–Mo(1)–Ge(1)	136.28(13)	C(7)–Mo(2)–Ge(2)	134.81(14)
C(4)–Mo(1)–Ge(1)	76.07(12)	C(8)–Mo(2)–Ge(2)	75.12(12)
C(2)–Mo(1)–C(3)	83.7(2)	C(6)–Mo(2)–C(7)	83.1(2)
C(3)–Mo(1)–C(4)	77.6(2)	C(7)–Mo(2)–C(8)	77.7(2)
C(4)–Mo(1)–C(2)	105.5(2)	C(8)–Mo(2)–C(6)	108.6(2)

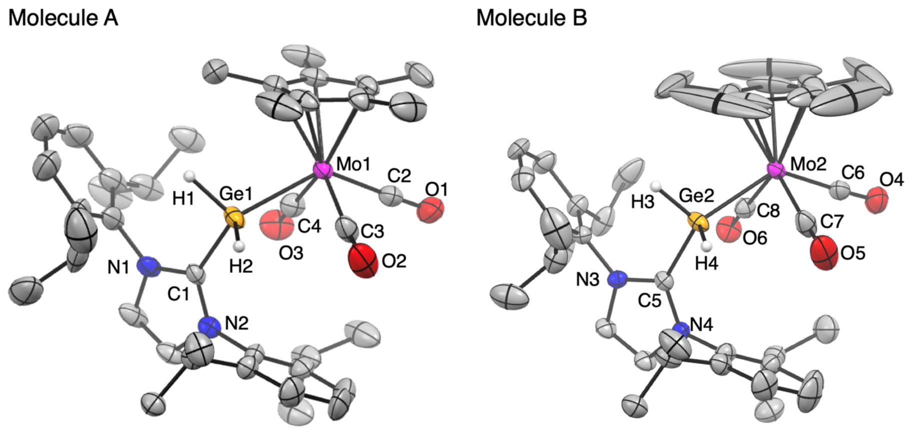

Figure S20. *Molecular structure of **3** in the crystalline state with thermal ellipsoids at 50% probability. Hydrogen atoms bound to carbon atoms and [B(C₆F₅)₄] counter anions are omitted for clarity.*

Table S3. Selected bond lengths [Å] and bond angles [°] in **3**.

Molecule A		Molecule B	
Bond lengths [Å]			
Mo(1)–Ge(1)	2.6065(6)	Mo(2)–Ge(2)	2.6099(6)
Ge(1)–C(1)	1.996(4))	Ge(2)–C(5)	1.999(4)
Ge(1)–H(1)	1.50(5)	Ge(2)–H(3)	1.44(5)
Ge(1)–H(2)	1.61(5)	Ge(2)–H(4)	1.54(5)
Mo(1)–C(2)	1.983(5)	Mo(2)–C(6)	1.984(5)
Mo(1)–C(3)	1.994(5)	Mo(2)–C(7)	1.990(5)
Mo(1)–C(4)	1.989(5)	Mo(2)–C(8)	1.994(5)
C(2)–O(1)	1.154(6)	C(6)–O(4)	1.149(5)
C(3)–O(2)	1.154(6)	C(7)–O(5)	1.145(6)
C(4)–O(3)	1.149(6)	C(8)–O(6)	1.147(6)
Bond angles [°]			
Mo(1)–Ge(1)–C(1)	120.94(13)	Mo(2)–Ge(2)–C(5)	121.24(11)
C(2)–Mo(1)–Ge(1)	76.85(15)	C(6)–Mo(2)–Ge(2)	76.11(13)
C(3)–Mo(1)–Ge(1)	134.32(14)	C(7)–Mo(2)–Ge(2)	134.23(13)
C(4)–Mo(1)–Ge(1)	73.19(14)	C(8)–Mo(2)–Ge(2)	72.25(14)
C(2)–Mo(1)–C(3)	78.4(2)	C(6)–Mo(2)–C(7)	78.31(19)
C(3)–Mo(1)–C(4)	78.8(2)	C(7)–Mo(2)–C(8)	80.1(2)
C(4)–Mo(1)–C(2)	107.7(2)	C(8)–Mo(2)–C(6)	107.3(2)

3. Theoretical calculations on 2 and 3

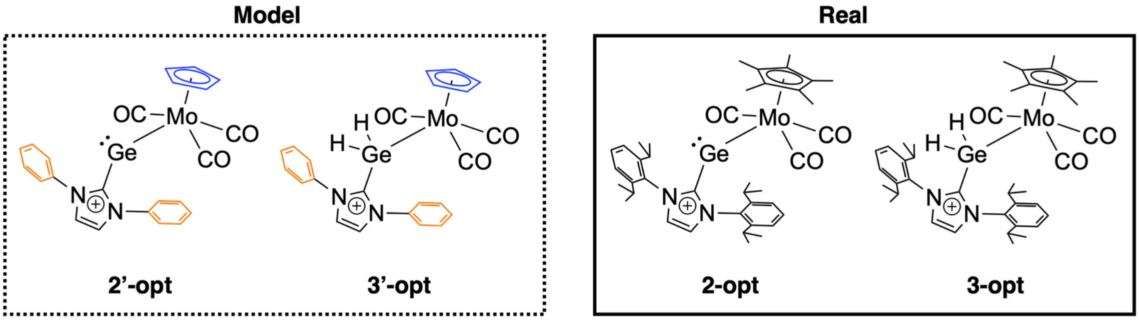

Chart S1. *Schematic representation of the model* ***(2′-opt****,* ***3′-opt****) and real (***2-opt****,* ***3-opt****) complexes used in the theoretical calculations.*

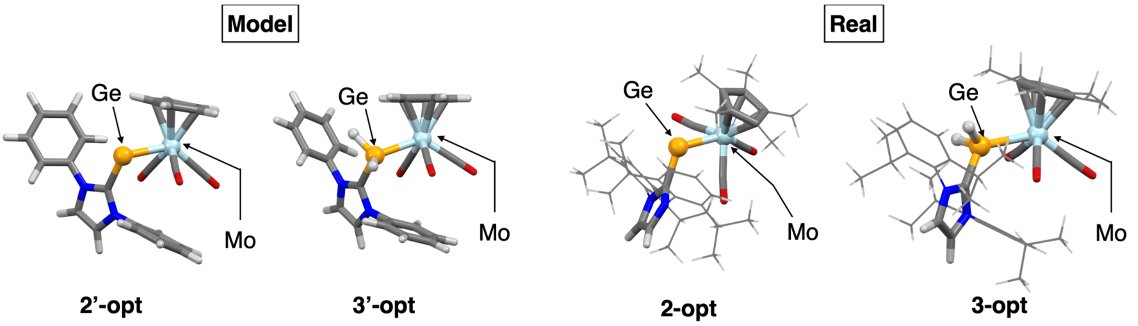

	Calc.		XRD		Calc.		XRD	
	**2'-opt**	**2-opt**	Complex **2**		**3'-opt**	**3-opt**	Complex **3**	
Ge–Mo [Å]	2.664	2.613	2.5701(6)	2.5713(6)	2.651	2.644	2.6065(6)	2.6099(6)
Ge–C(NHC) [Å]	2.077	2.069	2.048(4)	2.041(4)	1.997	2.016	1.996(4)	1.999(4)
Mo–C(O) [Å]	1.995	1.996	1.977(5)	1.987(5)	1.985	2.002	1.983(5)	1.984(5)
	1.999	2.009	1.987(5)	1.976(5)	2.015	2.009	1.989(5)	1.990(5)
	2.020	2.018	1.994(5)	1.995(5)	2.024	2.012	1.994(5)	1.994(5)
Mo–Ge–C(NHC) [°]	106.46	113.05	112.45(11)	112.88(11)	113.62	123.71	120.94(13)	121.24(11)

Figure S21. *Optimized structures of* **2** *and* **3***. Selected bond lengths [Å] and bond angles [°] for the model (***2′-opt****,* ***3′-opt****) and real (***2-opt****,* ***3-opt****) complexes.*

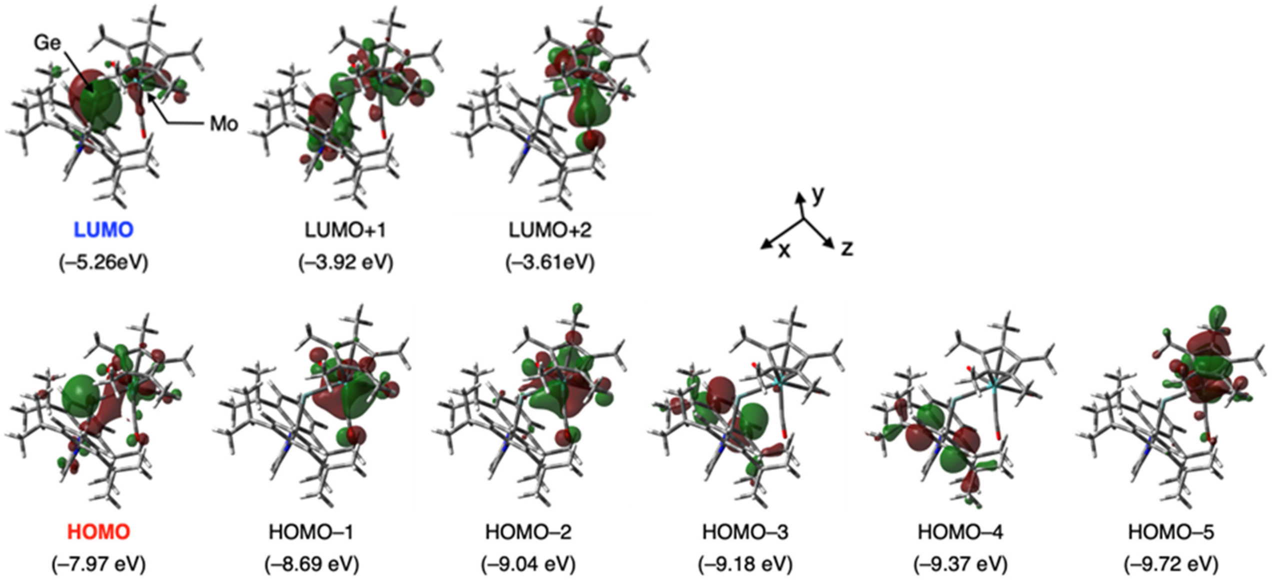

Figure S22. *Frontier molecular orbitals for* ***2-opt****.*

Table S4. Calculated singlet excited state for **2-opt**.

Excited State	Transition	Energy (Wavelength)	Oscillator Strength
S1	HOMO → LUMO (49.8%)	1.7885 eV (693.22 nm)	0.0129
S2	HOMO-1 → LUMO (49.2%)	2.4855 eV (498.84 nm)	0.0007
S3	HOMO-2 → LUMO (37.3%)	3.0098 eV (411.94 nm)	0.0014
	HOMO → LUMO+1 (10.4%)		
S4	HOMO-3 → LUMO (45.2%)	3.2924 eV (376.58 nm)	0.0072
	HOMO-2 → LUMO (2.2%)		
	HOMO → LUMO+1 (1.2%)		
	HOMO-5 → LUMO (1.0%)		
S5	HOMO → LUMO+2 (24.6%)	3.3724 eV (367.64 nm)	0.0382
	HOMO → LUMO+1 (12.2%)		
	HOMO-5 → LUMO (6.7%)		
	HOMO-2 → LUMO (2.6%)		
	HOMO-3 → LUMO (2.4%)		
S6	HOMO-5 → LUMO (23.9%)	3.4077 eV (363.83 nm)	0.0037
	HOMO → LUMO+2 (17.8%)		
S7	HOMO-6 → LUMO (44.9%)	3.4743 eV (356.86 nm)	0.0020
S8	HOMO → LUMO+1 (18.9%)	3.5809 eV (346.24 nm)	0.2492
	HOMO-5 → LUMO (13.0%)		

-

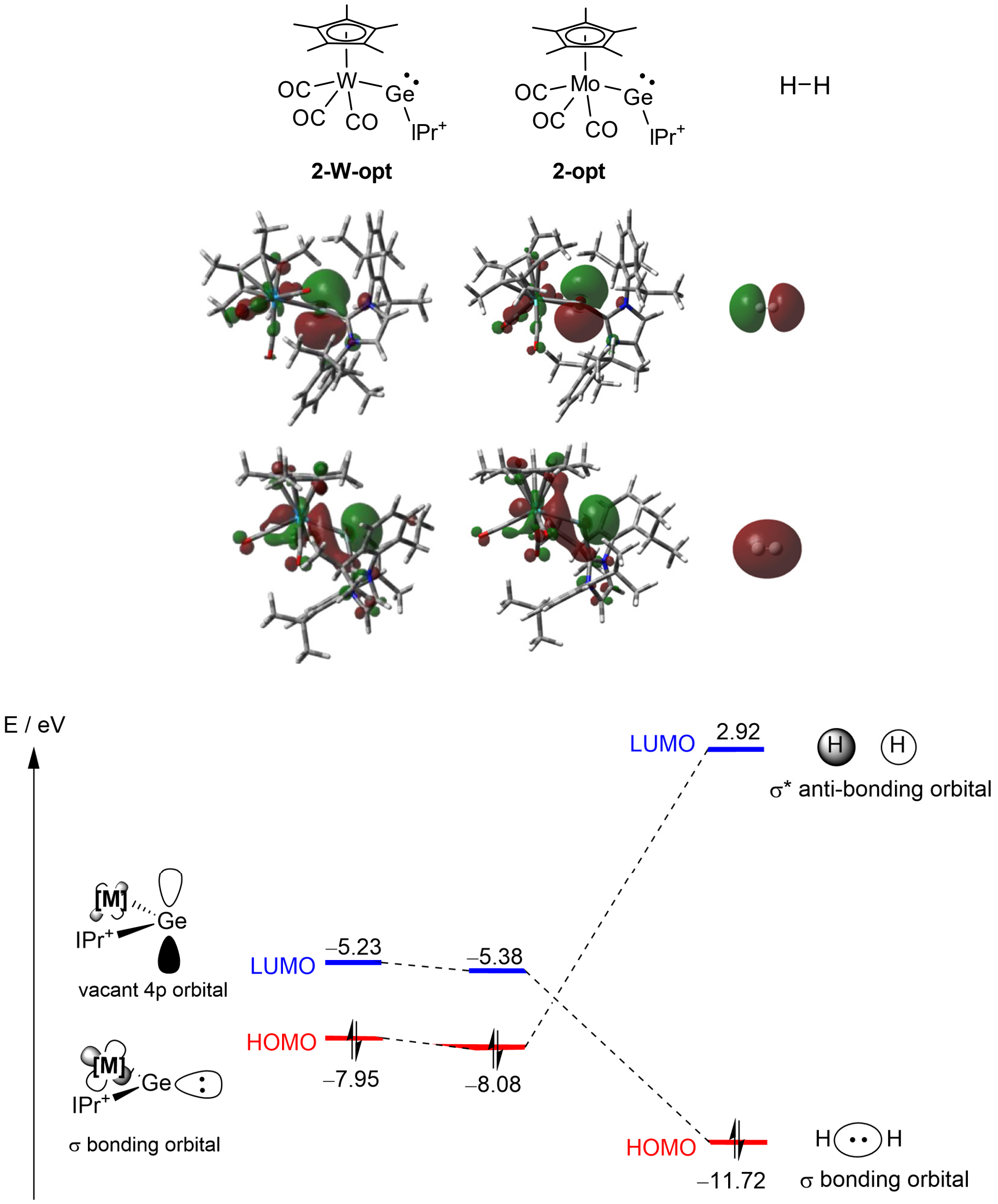

Figure S23. *Energy levels of the frontier molecular orbitals of* ***2-W-opt****,* ***2-opt****, and* H_2*.*

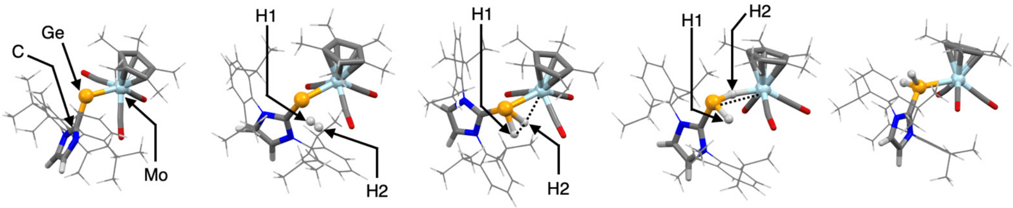

	2-opt (0 kJ/mol)	INT1 (22.1)	TS1 (110.0) 1448.5*i* cm^{-1}	INT2 (45.2)	3-opt (–75.2)
Ge–Mo	2.613	2.621	2.891	3.390	2.644
H1–H2		0.747	1.127	2.515	2.487
Ge–H1		3.112	1.684	1.574	1.533
Ge–H2		3.756	1.786	1.732	1.545
Mo–H1		4.712	3.312	3.519	3.578
Mo–H2		5.109	2.288	1.877	3.515
C–Ge	2.069	2.085	2.090	2.066	2.016
C–Ge–Mo	113.05	111.21	108.04	104.22	123.71
C–Ge–H1				92.89	
C–Ge–H2				91.99	
Ge–H2–Mo				139.88	

Figure S24. *Comparison of the main structural parameters of DFT-optimized* ***2-opt***, ***3-opt***, *intermediates* ***INT1*** *and* ***INT2***, *as well as transition state* ***TS1***, *calculated at the SMD(C_6H_5F)/M06/LANL2DZ[Mo,W]:6-31+G(d,p)[H,C,N,O,Ge] level.*

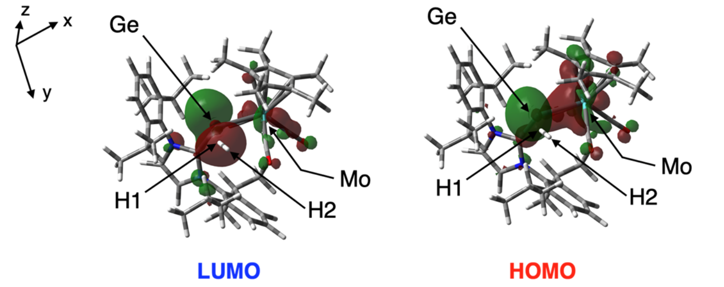

Figure S25. *HOMO and LUMO of* ***INT-1***.

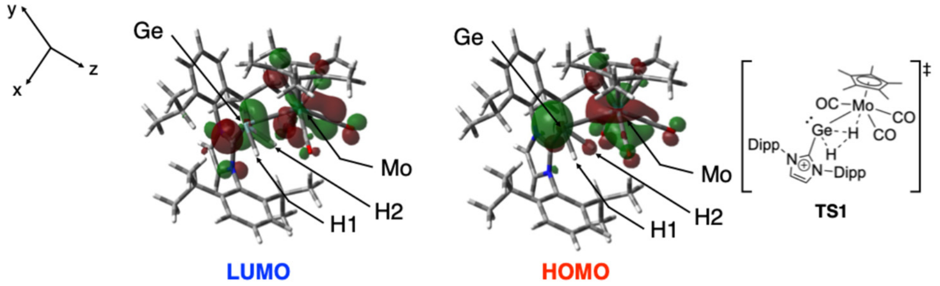

Figure S26. *HOMO, LUMO and schematic description of* ***TS-1***.

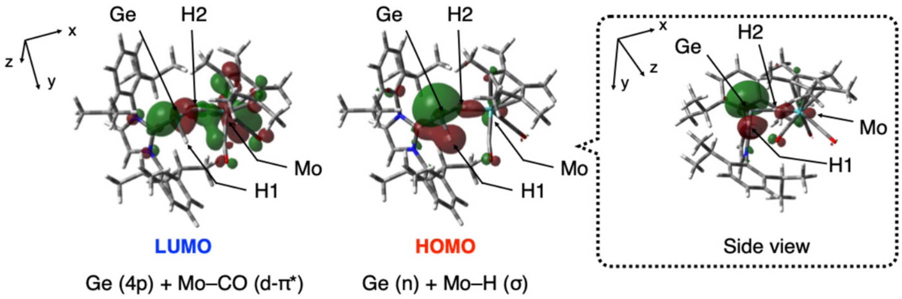

Figure S27. *HOMO and LUMO of **INT-2**.*
