# Supplementary material for: Metal–Ligand Cooperation in Dihydrogen Activation by a Cationic Metallogermylene: Enhanced Activity from Tungsten to Molybdenum
Source: Molecules. 2024 Dec 18;29(24):5974. doi: 10.3390/molecules29245974 (PMC11676882; doi:10.3390/molecules29245974)
Supplement: Supplementary file 1 [file molecules-29-05974-s001.zip › MoGe_Mol_ESI_2_xyz_P21-30.pdf]

4．Atomic coordinates for the computed complexes and transition states

Model structures

2′-opt: E(RM06) = -3363.04372938

Ge	0.65858799	0.21772927	-1.17301515
C	-2.09190529	0.25468434	-1.12599478
O	-2.63201844	-0.35072332	-1.95338449
C	-2.75651567	0.81647239	1.34114851
O	-3.61043201	0.42309549	2.00455856
C	-0.28474325	0.39472358	1.55408669
O	0.22340581	-0.18460461	2.42428052
C	1.16526685	-1.45394860	-0.04854719
N	0.49691164	-2.56264797	0.32553488
C	1.31902678	-3.39714525	1.06468501
H	0.96246754	-4.34109597	1.45260666
C	2.53009275	-2.79244805	1.14093136
H	3.44562260	-3.08087016	1.63764405
N	2.41852766	-1.60184217	0.44209277
C	-0.86738578	-2.86127994	-0.01326393
C	-1.82490279	-2.88332416	0.99677341
C	-3.13707106	-3.20720577	0.66552598
H	-3.89937124	-3.22150488	1.43999267
C	-3.47163375	-3.51025852	-0.65383072
H	-4.49766815	-3.76483856	-0.90562628
C	-2.49913164	-3.49059090	-1.65021228
H	-2.76347017	-3.72717207	-2.67713741
C	-1.18318519	-3.16622578	-1.33349047
C	3.45745560	-0.64324061	0.23720511
C	4.68897466	-1.06308992	-0.25527567
C	5.67608863	-0.11364208	-0.49858327
H	6.64029588	-0.43029282	-0.88674521
C	5.42652621	1.23902803	-0.26740048
H	6.20151677	1.97425975	-0.46563293
C	4.19184715	1.64658262	0.22674429
H	4.00654297	2.69761624	0.43348282
C	3.20012291	0.70361196	0.49490356
C	0.10559424	3.34990212	-0.42949902
C	-0.47584929	3.66349982	0.83535971
C	-1.87914305	3.73995338	0.65825422
C	-2.17843978	3.46875673	-0.70805918
C	-0.94741944	3.22838729	-1.38198619
Mo	-1.24348921	1.49446199	0.18684393
H	-2.60247553	3.96579499	1.43352727
H	-3.16298454	3.47766045	-1.15983725
H	-0.82811969	3.02699200	-2.44116163
H	1.16737570	3.28473149	-0.64454439
H	0.05869432	3.83626329	1.76202411
H	-1.54138223	-2.64306150	2.02030858
H	-0.40397334	-3.16397519	-2.09430846
H	2.26965418	1.00079950	0.97858391
H	4.86126921	-2.11597225	-0.46801502

INT1′: E(RM06) = -3364.21636813

Ge	-0.78308768	-0.23428728	-1.07441368
C	2.01356638	-0.27372472	-1.07760850
O	2.57663397	0.39362048	-1.83974708
C	2.69444569	-1.04553356	1.32046612
O	3.57167275	-0.73686618	1.99816703
C	0.25017610	-0.50424877	1.56289476
O	-0.22578434	0.07091705	2.45612849
C	-1.06011442	1.54194330	-0.01709219
N	-0.33694129	2.62436515	0.33514626
C	-1.12443654	3.52573601	1.03199740
H	-0.72046752	4.45976722	1.39690357
C	-2.36769070	2.98849274	1.11205100
H	-3.27582458	3.34047966	1.58090142
N	-2.30728924	1.77256466	0.45656107
C	1.05827195	2.84165394	0.05102682
C	1.99567261	2.57969785	1.04561282
C	3.33909869	2.82798535	0.77920038
H	4.08535948	2.62660123	1.54326694
C	3.72363584	3.33516359	-0.46090419
H	4.77398442	3.52767183	-0.66291761
C	2.77024403	3.60202350	-1.44069629
H	3.07357037	4.00272313	-2.40408851
C	1.42363532	3.36011346	-1.18619760
C	-3.38353422	0.84061714	0.28502771
C	-4.46552663	1.19226635	-0.51554509
C	-5.47230602	0.25682493	-0.73137784
H	-6.32067563	0.51475687	-1.35936054
C	-5.38708149	-1.01200617	-0.15810351
H	-6.17542251	-1.73859432	-0.33552746
C	-4.30443571	-1.34638673	0.65033901
H	-4.25359435	-2.32615787	1.11849270
C	-3.29340233	-0.41542802	0.88534068
C	-0.31961815	-3.37887399	-0.38585946
C	0.42011936	-3.77652655	0.76654575
C	1.78458566	-3.87080402	0.38968434
C	1.90101024	-3.53184056	-0.98635347
C	0.59680490	-3.22481065	-1.46780681
H	0.66090959	1.41789421	-3.29266495
H	-0.04204786	1.33311253	-3.53277573
H	0.33953669	-2.96319997	-2.48872277
H	2.81415880	-3.53195921	-1.56915626
H	2.60214174	-4.15294264	1.04359961
H	0.01340753	-3.99517468	1.74651936
H	-1.39804060	-3.26900750	-0.44316882
H	-2.47506604	-0.63346066	1.56952051
H	-4.50193892	2.17680032	-0.97720178
H	0.65991857	3.57552807	-1.93109956
H	1.67067434	2.19338611	2.01049773
Mo	1.14045080	-1.58892661	0.14747691

TS1′: E(RM06) = -3364.18545074

Ge	0.28753303	0.29994840	-1.53407679
C	0.11813054	-2.80089490	-1.09912711
O	0.73360649	-3.41503400	-1.85629104
C	-0.55472457	-3.22098877	1.37058456
O	-0.32099736	-4.09420205	2.08221129
C	0.03347103	-0.71212554	1.52817301
O	0.57645035	-0.12338505	2.36421503
C	1.01846193	1.60726979	-0.07867570
N	2.24556550	1.71131229	0.48114629
C	2.26708752	2.71838863	1.43018727
H	3.16569100	2.95379788	1.98286265
C	1.03041783	3.27075942	1.44423787
H	0.61862448	4.09803767	2.00475512
N	0.28184586	2.58409950	0.50244518
C	3.38278649	0.88150946	0.18707393
C	3.81709038	-0.02593225	1.14769005
C	4.91866954	-0.82808871	0.86071600
H	5.26950011	-1.54477339	1.59839392
C	5.56504883	-0.71431752	-0.36815714
H	6.42304073	-1.34413987	-0.58786361
C	5.12117303	0.20696031	-1.31545225
H	5.63277399	0.29883550	-2.26965906
C	4.02469123	1.01719878	-1.03958675
C	-1.09322165	2.86511437	0.21652746
C	-1.46678154	3.23698143	-1.07257098
C	-2.80564040	3.51982139	-1.33227758
H	-3.10778208	3.81674662	-2.33308747
C	-3.74820345	3.44780060	-0.30809505
H	-4.78948709	3.68183611	-0.51312783
C	-3.35653518	3.09179319	0.98167039
H	-4.09111210	3.04559905	1.78178139
C	-2.02378422	2.79168363	1.24967714
C	-2.78575079	-1.47782915	-1.40865221
C	-2.82026826	-0.29884450	-0.60535465
C	-3.10982381	-0.68389496	0.73050265
C	-3.26314718	-2.09393867	0.75788615
C	-3.07456436	-2.58927495	-0.56673701
H	0.99643575	-1.03518353	-0.53223064
H	1.70444654	-0.57623755	-1.28180330
Mo	-1.02734993	-1.70490739	0.13694604

H	-3.15903781	-3.62371189	-0.87790071
H	-3.50654178	-2.68929552	1.63051611
H	-3.20982104	-0.01434481	1.57717091
H	-2.69590806	0.71995345	-0.95815434
H	-2.61398552	-1.51299501	-2.47859825
H	-1.70106345	2.49913238	2.24819207
H	-0.71409869	3.33212885	-1.85312502
H	3.66554155	1.74640952	-1.76322339
H	3.28857045	-0.10585416	2.09616186

TS2′: E(RM06) = -3364.14234530

Ge	0.40271308	-0.04182138	-1.29339265
C	-2.44513608	0.18040232	-0.95929608
O	-3.12123918	-0.47268140	-1.62575405
C	-2.72487959	0.87265497	1.49137211
O	-3.50298128	0.52106303	2.26430852
C	-0.17634063	0.58173685	1.50269863
O	0.50640842	0.12977015	2.32180707
C	1.30890365	-1.43388938	-0.14418271
N	0.65358941	-2.51161327	0.34158356
C	1.47304360	-3.23467219	1.19284147
H	1.12850682	-4.13915520	1.67362865
C	2.66944815	-2.60075142	1.21256202
H	3.59703276	-2.82893525	1.71815187
N	2.55442329	-1.50193107	0.37668651
C	-0.70885456	-2.86286269	0.05307942
C	-1.63720377	-2.85528274	1.09062666
C	-2.95061697	-3.22677929	0.81967197
H	-3.68700823	-3.22048530	1.61902006
C	-3.31882267	-3.59924484	-0.47197276
H	-4.34581360	-3.88745603	-0.67962746
C	-2.37780941	-3.60246377	-1.49897880
H	-2.66741552	-3.89651829	-2.50407466
C	-1.05935893	-3.23848270	-1.24086988
C	3.59907695	-0.54059018	0.16802373
C	4.24049521	-0.48657004	-1.06481340
C	5.23592531	0.46643235	-1.26036816
H	5.74856951	0.52237889	-2.21692897
C	5.58239912	1.33678004	-0.22786165
H	6.36442467	2.07527918	-0.38393586
C	4.93919292	1.25875390	1.00610192
H	5.21715849	1.93447814	1.81061473
C	3.93643271	0.31515579	1.21197672
C	-0.17196116	3.50724524	0.28513393
C	-1.44548982	3.67277552	0.89415086
C	-2.44234379	3.51665277	-0.12234277
C	-1.77809142	3.26381909	-1.35313730
C	-0.37915069	3.25607033	-1.10150860
Mo	-1.35336761	1.47280926	0.14791454
H	-3.51282078	3.61571098	0.01343382
H	-2.25163093	3.11614175	-2.31666730
H	0.39619694	3.11692844	-1.84759894
H	0.79033319	3.57579925	0.77985920
H	-1.62917199	3.91024111	1.93517436
H	-1.33145405	-2.55590967	2.09215677
H	-0.30517654	-3.26205599	-2.02543326
H	3.40542635	0.24579492	2.15988233
H	3.96065526	-1.18239947	-1.85370587
H	1.61819845	1.02604778	-0.62150895
H	1.73186891	0.70266616	-1.78451299

INT2′: E(RM06) = -3364.20696304

Ge	-0.90487735	0.32017711	-1.74088774
C	2.37377259	-0.14516251	-1.01701239
O	3.05225059	0.53841208	-1.64410804
C	2.77690559	-0.84213970	1.39759327
O	3.54713750	-0.41793601	2.14010975
C	0.23607814	-0.73647426	1.52602063
O	-0.44384812	-0.32472644	2.36494695
C	-1.33596640	1.50193905	-0.09673474
N	-0.66053170	2.49932646	0.52520502
C	-1.37425260	2.96592929	1.61288264
H	-1.00088141	3.77003762	2.23145075
C	-2.52664251	2.25132969	1.66284681
H	-3.37259550	2.29664238	2.33409464
N	-2.48777415	1.36684684	0.60421011
C	0.66238713	2.96033753	0.20220401
C	1.69141380	2.69561200	1.10152159
C	2.97693282	3.13984130	0.80496415
H	3.78982519	2.93775920	1.49792414
C	3.21766260	3.83428004	-0.37861897
H	4.22289811	4.17612262	-0.61031445
C	2.17473608	4.09829309	-1.26436295
H	2.36429823	4.64820178	-2.18210074
C	0.88448134	3.66505079	-0.97507121
C	-3.51238570	0.39281246	0.33900372
C	-4.30712937	0.52054212	-0.79568148
C	-5.29438065	-0.43032243	-1.03419416
H	-5.92266988	-0.34366772	-1.91645411
C	-5.48303620	-1.48253237	-0.14018280
H	-6.25988930	-2.21887456	-0.32864638
C	-4.68705161	-1.58933550	0.99830632
H	-4.84212116	-2.40591030	1.69862055
C	-3.69117716	-0.64810214	1.24487565
C	0.40428820	-3.67814050	0.26766360
C	1.70476050	-3.72012831	0.84398569
C	2.65507526	-3.48029109	-0.20164000
C	1.93662516	-3.29862666	-1.41536826
C	0.55242481	-3.42112952	-1.12441459
H	-0.08747271	-0.87601691	-0.76764076
H	0.36169932	1.18647929	-2.09518970
Mo	1.39773450	-1.55065578	0.10345205
H	-0.53280233	-3.84163993	0.78649362
H	-0.25712449	-3.32077138	-1.83938927
H	2.36821724	-3.11372093	-2.39195596
H	3.73357469	-3.48107627	-0.09563294
H	1.93803662	-3.93907770	1.87912545
H	-4.15682063	1.35742003	-1.47458466
H	-3.05485280	-0.71417605	2.12543104
H	0.05680848	3.86953752	-1.65067046
H	1.48378886	2.14390188	2.01784663

3′-opt: E(RM06) = -3364.26153910

Ge	-0.37929133	0.12910357	-1.25542584
C	2.27888034	-0.67790580	-0.96523437
O	3.06400687	-0.16416788	-1.63641482
C	2.46469339	-1.49964422	1.45689148
O	3.32540788	-1.36408364	2.20804268
C	0.13256660	-0.49240673	1.49653485
O	-0.35872910	0.16748411	2.31704021
C	-0.91892620	1.72310586	-0.17941653
N	-0.09820811	2.66794758	0.32199764
C	-0.78525292	3.47871964	1.20613175
H	-0.30081714	4.30571321	1.70582102
C	-2.06353843	3.02520655	1.24095912
H	-2.93486665	3.36777498	1.78092219
N	-2.12756623	1.94827474	0.37635450
C	1.31324214	2.76249758	0.05897091
C	2.20241579	2.46159246	1.08745011
C	3.56855937	2.55172118	0.83893447
H	4.27579125	2.31337823	1.62909566
C	4.02468143	2.93957511	-0.41968509
H	5.09251333	3.00595869	-0.61026054
C	3.12057355	3.24478685	-1.43467981
H	3.48073883	3.55252103	-2.41244508
C	1.75148031	3.16105719	-1.19933222
C	-3.29143876	1.12251334	0.18864396
C	-4.03910230	1.24619992	-0.97688127
C	-5.14299547	0.41770510	-1.15404547
H	-5.73845418	0.49939911	-2.05929843
C	-5.48585068	-0.50946875	-0.17109672
H	-6.35111221	-1.15166820	-0.31344523
C	-4.73262476	-0.61025147	0.99715860
H	-5.01033572	-1.32641716	1.76619049

C	-3.62325936	0.21011177	1.18504081
C	-0.88915362	-3.11184804	-0.17547308
C	-0.08920500	-3.69601106	0.84544125
C	1.16040663	-4.05713260	0.26349046
C	1.12899887	-3.72445452	-1.11498777
C	-0.13230188	-3.13025628	-1.39045516
Mo	0.93880627	-1.69823515	0.14112792
H	1.98965080	-4.52509984	0.78190306
H	1.92655931	-3.89125095	-1.82927619
H	-0.48289413	-2.80673605	-2.36449978
H	-1.90725671	-2.75249555	-0.06781328
H	-0.37967609	-3.85428738	1.87696651
H	1.82377294	2.15518161	2.06163311
H	1.02926509	3.40625436	-1.97527373
H	-3.01435581	0.14726359	2.08531427
H	-3.75151489	1.97506159	-1.73192699
H	-1.74996897	-0.41693944	-1.70778962
H	0.32826111	0.75105488	-2.47353422

Real structures

H_2: E(RM06) = -1.16958422959

H	-0.00000000	-0.00000000	0.37107800
H	-0.00000000	-0.00000000	-0.37107800

2 (M = Mo): E(RM06) = -4031.37279875

Mo	-2.24255430	0.33251286	-0.01641768
Ge	0.09077210	-0.30221217	-1.00496832
C	3.23692231	-2.12599350	-1.75854816
H	3.25544299	-1.07259178	-1.44239203
C	2.77895536	-2.15165367	-3.21411494
H	2.87213046	-3.15175668	-3.65537591
H	3.40570445	-1.47890238	-3.81267376
H	1.73561685	-1.82589064	-3.31885997
C	4.66764562	-2.65738673	-1.63300283
H	5.03225318	-2.60892748	-0.59957751
H	5.34980260	-2.07303813	-2.26227627
H	4.72359951	-3.70490187	-1.95611903
C	2.01260168	2.43949461	0.24341750
C	1.52561751	3.29933120	1.23817654
C	1.23188801	4.60952483	0.85296774
H	0.85164511	5.31066592	1.59308048
C	1.41732438	5.03038813	-0.45693436
H	1.18290545	6.05523556	-0.73401402
C	1.89550320	4.14872073	-1.41940846
H	2.02519349	4.49417898	-2.44218328
C	2.20394157	2.82757514	-1.09596273
C	1.35146106	2.87008279	2.67990582
H	1.42087612	1.77370735	2.72986756
C	-0.01885949	3.25382489	3.23328768
H	-0.12269600	4.33963183	3.34990962
H	-0.16512981	2.80310795	4.22152431
H	-0.82962274	2.90826379	2.58123753
C	2.46283231	3.45815252	3.55204722
H	3.46186335	3.17156036	3.20051601
H	2.35788321	3.11934271	4.58938174
H	2.41894113	4.55495476	3.55201460
C	2.77540509	1.88959412	-2.14299244
H	2.51159346	0.85719053	-1.86730157
C	2.20103551	2.11997913	-3.53783374
H	1.10361567	2.14996298	-3.52932734
H	2.51552017	1.31024050	-4.20718551
H	2.56021807	3.05746788	-3.97997343
C	4.30329782	1.96789481	-2.16549510
H	4.63339963	2.97599302	-2.44859821
H	4.71361084	1.25960635	-2.89719062
H	4.74185517	1.73467529	-1.18685572
O	-1.22848136	2.82452473	-1.65434161
O	-3.46991951	2.48437247	1.96182477
O	-0.42943274	0.06811569	2.56767673
N	2.35901821	-1.06034563	0.77697948
N	2.36920406	1.09087994	0.61948629
C	-3.23759704	-0.50062531	-2.00360802
C	-4.24445119	0.14941659	-1.22025392
C	-4.41713661	-0.61214708	-0.00913513
C	-3.50377103	-1.70290731	-0.03494180
C	-2.78317514	-1.64902009	-1.27604794
C	-2.82932689	-0.13365668	-3.39280086
H	-1.75747270	-0.29364426	-3.56676901
H	-3.04417244	0.91690032	-3.61397743
H	-3.37148824	-0.74293718	-4.12755709
C	-5.07711460	1.31312654	-1.65153391
H	-5.94792958	0.97637834	-2.22909690
H	-4.51173914	2.00747907	-2.28259839
H	-5.45313223	1.88472343	-0.79629002
C	-5.46017976	-0.38425388	1.03453667
H	-5.79356434	0.65782500	1.06734635
H	-5.10115579	-0.64872852	2.03514168
H	-6.34300622	-1.00300321	0.82699540
C	-3.42437430	-2.80903753	0.96339348
H	-3.63346893	-2.45840069	1.98020716
H	-2.43315909	-3.27860162	0.96933937
H	-4.15698471	-3.59380943	0.72919681
C	-1.92124024	-2.74538516	-1.81264940
H	-1.29377189	-3.20452681	-1.03619074
H	-1.26225955	-2.40708999	-2.62055064
H	-2.55493256	-3.54021532	-2.22930878
C	-1.53240116	1.92562304	-0.98572538
C	-2.99209245	1.71553340	1.24904948
C	-1.03471431	0.12439551	1.57642953
C	1.64405316	-0.00521542	0.32882542
C	3.53411069	-0.62695754	1.36803332
H	4.24709779	-1.31721129	1.79722948
C	3.54071942	0.72619457	1.26702043
H	4.26012343	1.46629361	1.59004278
C	1.98014476	-2.41637340	0.45650894
C	1.26974735	-3.17330950	1.40190020
C	0.83414223	-4.43849687	0.99822312
H	0.27995990	-5.06031737	1.69855211
C	1.11534911	-4.92323880	-0.27407920
H	0.76630188	-5.91175439	-0.56389655
C	1.86677118	-4.16800730	-1.16788601
H	2.10887036	-4.57746244	-2.14645384
C	2.32615782	-2.89602493	-0.82268653
C	1.08386134	-2.70986674	2.83001605
H	1.21611540	-1.62032737	2.86230918
C	-0.30088204	-3.01654716	3.39011395
H	-1.09581518	-2.63280731	2.73971882
H	-0.41823077	-2.54480512	4.37210225
H	-0.45918211	-4.09413227	3.52589598
C	2.16530687	-3.34420430	3.71054124
H	2.07958402	-2.98398168	4.74229438
H	2.06335545	-4.43727154	3.72521470
H	3.17664644	-3.11420462	3.35294381

INT1 (M = Mo): E(RM06) = -4032.54502042

Ge	-0.21039872	-0.58746816	-0.94147667
C	1.75938838	1.23251592	-1.39389042
O	1.59771151	2.00218384	-2.24931358
C	3.13403387	1.34883073	0.84959547
O	3.69434518	2.21301861	1.36751945
C	1.05516759	-0.03697522	1.52654396
O	0.46795789	0.03954006	2.52756817
C	-1.59816005	0.36150760	0.29197327
N	-1.89933208	1.63091898	0.63901338
C	-3.09038660	1.65727379	1.34849790
H	-3.50976004	2.58610900	1.70960608
C	-3.53280188	0.37827704	1.43635676
H	-4.40936434	-0.05154990	1.90160556
N	-2.60757398	-0.40107969	0.76861646
C	-1.14687923	2.81096011	0.27481448
C	-0.47410080	3.50736873	1.29073730
C	0.23820468	4.64853063	0.91166587
H	0.78067307	5.21358952	1.66674812
C	0.26089039	5.07306815	-0.40821394

H	0.82325175	5.96251889	-0.68160821
C	-0.43624984	4.37155902	-1.38578852
H	-0.40148622	4.72233652	-2.41303047
C	-1.16005330	3.22148916	-1.07374894
C	-2.74210013	-1.80518623	0.46399306
C	-3.31679359	-2.14541014	-0.77627284
C	-3.40225831	-3.50292677	-1.08095982
H	-3.83543204	-3.81192282	-2.03000089
C	-2.94446136	-4.46796553	-0.18889223
H	-3.02441969	-5.52138932	-0.44680883
C	-2.39834991	-4.09674489	1.03264172
H	-2.06031835	-4.86555496	1.72510142
C	-2.28409784	-2.75054180	1.39252749
C	2.49070957	-2.50327344	-0.68844010
C	3.35148612	-2.24566060	0.43278949
C	4.33726454	-1.30238172	0.02122126
C	4.08012293	-0.94336503	-1.34314280
C	2.93982595	-1.69371488	-1.78401822
H	-0.11195194	0.23890784	-4.60372531
H	-0.36207047	-0.15243983	-4.01921269
C	-3.85545987	-1.10255672	-1.73439937
H	-3.40970241	-0.13062869	-1.47670538
C	-3.49033957	-1.38528472	-3.18986819
H	-4.05098354	-2.23692749	-3.59509732
H	-3.73468323	-0.51534659	-3.81275018
H	-2.41986325	-1.59767130	-3.30585073
C	-5.37094577	-0.96420284	-1.57257908
H	-5.87583796	-1.90560160	-1.82538216
H	-5.65171748	-0.70176932	-0.54502286
H	-5.75837934	-0.18423778	-2.23955869
C	-1.73483714	-2.36417869	2.74869168
H	-1.59688297	-1.27427367	2.77526526
C	-0.36741790	-2.99071805	3.00674853
H	0.33907998	-2.72779080	2.20838208
H	0.04875001	-2.62351850	3.95215698
H	-0.42141406	-4.08538273	3.06911466
C	-2.72835902	-2.73731110	3.85017203
H	-3.70680725	-2.26780740	3.68950879
H	-2.88588419	-3.82280292	3.89020098
H	-2.35398208	-2.41698644	4.82927719
C	-1.95260162	2.48651381	-2.14434892
H	-1.77298800	1.40097397	-2.02836073
C	-3.45790949	2.72434933	-1.98403642
H	-3.84526846	2.40820292	-1.00847970
H	-3.68202838	3.79245740	-2.10358493
H	-4.01437479	2.17760113	-2.75692209
C	-1.54040660	2.83848442	-3.56878836
H	-2.05926566	2.17627632	-4.27200971
H	-1.82634604	3.86636154	-3.82653443
H	-0.46233360	2.72829765	-3.72977802
C	-0.54045824	3.11397030	2.75336373
H	-0.93059008	2.08888309	2.82408824
C	0.82729363	3.11684138	3.43262682
H	0.73886739	2.70804621	4.44577069
H	1.55910145	2.50978951	2.88983060
H	1.23532685	4.13082197	3.52549843
C	-1.49312619	4.04642671	3.50755427
H	-1.58597964	3.73600491	4.55495861
H	-1.11517932	5.07688900	3.49653961
H	-2.49844131	4.06778121	3.06892525
C	2.42626965	-1.74617680	-3.18525430
H	2.98909884	-2.48470361	-3.77096340
H	1.36942566	-2.03489184	-3.23076606
H	2.52462809	-0.77986376	-3.69214061
C	1.45996193	-3.58422721	-0.75694301
H	0.90630334	-3.69134422	0.18469469
H	0.72583223	-3.41507934	-1.55332807
H	1.94097549	-4.54937871	-0.96453917
C	3.34439638	-2.96606086	1.74147097
H	2.40364244	-3.50023315	1.90746913
H	4.15152101	-3.71038396	1.77364624
H	3.49353007	-2.28617169	2.58821157

C	5.50675265	-0.86974720	0.84133160
H	6.31674897	-1.60630150	0.75663427
H	5.90768713	0.09505648	0.51416395
H	5.25292268	-0.78353503	1.90351628
C	4.94047095	-0.06889336	-2.19584775
H	5.75372724	-0.64911308	-2.65148320
H	4.37261288	0.39373218	-3.01003524
H	5.39906540	0.74014829	-1.61679713
Mo	2.23761522	-0.19692371	-0.08885935

TS1 (M = Mo): E(RM06) = -4032.50992228

Ge	0.27360027	-0.06562470	-1.24609017
C	-1.73860622	-2.63929104	-0.88981976
O	-1.50999238	-3.65050701	-1.40476126
C	-3.00014020	-2.09719698	1.27990049
O	-3.45817487	-2.79272204	2.07837803
C	-1.12600586	-0.42566744	1.49757423
O	-0.51970742	-0.22665906	2.46502044
C	1.60353225	0.28590959	0.32671544
N	2.56023971	-0.52735407	0.83356273
C	3.38650484	0.17147180	1.69519339
H	4.21516300	-0.31011048	2.19600946
C	2.94255430	1.45084242	1.71207431
H	3.29721293	2.32752701	2.23610390
N	1.84927116	1.50679832	0.86253903
C	2.84653630	-1.86546234	0.37212828
C	2.33883273	-2.95989308	1.08337635
C	2.58851114	-4.22792849	0.55269736
H	2.20666385	-5.10722189	1.06780923
C	3.31980144	-4.38483782	-0.61810698
H	3.49610279	-5.38148904	-1.01528694
C	3.84281554	-3.27667609	-1.27512587
H	4.43370597	-3.41746930	-2.17835856
C	3.62180600	-1.98645362	-0.79327183
C	1.16116849	2.73153189	0.52661836
C	1.37012319	3.27724569	-0.75770853
C	0.70255880	4.46557435	-1.05755138
H	0.83067879	4.91757120	-2.03820311
C	-0.09433746	5.10324014	-0.11217106
H	-0.59114520	6.03694439	-0.36524074
C	-0.23717096	4.56492038	1.15797392
H	-0.84571144	5.08570541	1.89508587
C	0.38224167	3.36176599	1.51070692
C	-3.52956494	-0.41945957	-2.07590396
C	-2.90877961	0.83039366	-1.77012038
C	-3.35275211	1.25000331	-0.47910038
C	-4.28470351	0.28171357	0.00666135
C	-4.40138191	-0.75621876	-0.98908450
H	-0.14228056	-1.63669430	-0.50623899
H	0.78028674	-1.66886631	-1.15284896
Mo	-2.26062310	-0.86875845	-0.11215077
C	-3.41382983	-1.14239243	-3.37733687
H	-4.05125621	-0.67075382	-4.13694617
H	-2.38616706	-1.13214675	-3.75960816
H	-3.72460983	-2.18862702	-3.29318814
C	-5.37672364	-1.88809197	-0.98308568
H	-5.69908170	-2.15006044	0.02962682
H	-6.27549781	-1.61813519	-1.55253774
H	-4.95804590	-2.79241381	-1.43860354
C	-5.11289570	0.42367923	1.24212628
H	-5.96793623	1.08665067	1.05612768
H	-5.51289730	-0.53600989	1.58408381
H	-4.53661089	0.85140271	2.07060015
C	-3.04834001	2.57821741	0.12591998
H	-3.58975662	3.37097190	-0.40931452
H	-3.34953220	2.63010611	1.17702567
H	-1.98046675	2.82162322	0.07112272
C	-2.15131256	1.67753335	-2.73314411
H	-2.86417392	2.28646713	-3.30765846
H	-1.46346108	2.36869183	-2.23075883
H	-1.57782075	1.08482243	-3.45439727
C	1.59596094	-2.80900506	2.39256881

H	1.36452844	-1.74545236	2.53975373
C	4.23494247	-0.79385180	-1.50046668
H	3.84843371	0.12607427	-1.03776704
C	3.86760318	-0.73778650	-2.98218557
H	2.78002199	-0.76195087	-3.13359159
H	4.25625218	0.18534610	-3.43186792
H	4.30083368	-1.57714195	-3.54000022
C	5.75254935	-0.78235247	-1.31084964
H	6.21597474	-1.66058125	-1.77802272
H	6.19055994	0.11181481	-1.77124630
H	6.02656849	-0.78859345	-0.24860267
C	0.27210196	-3.57016350	2.40322426
H	-0.29699771	-3.32852793	3.30928964
H	-0.34772142	-3.31327584	1.53380672
H	0.42542584	-4.65633339	2.39359539
C	2.48345882	-3.25397073	3.55715338
H	1.96599407	-3.10686633	4.51239279
H	2.73667892	-4.31874277	3.47345964
H	3.42586721	-2.69316379	3.58995966
C	2.37024068	2.69774978	-1.74276313
H	2.42677189	1.60942439	-1.59834597
C	1.99822331	2.92410156	-3.20435682
H	2.67114889	2.34788346	-3.85012210
H	0.97076962	2.60655462	-3.42236701
H	2.10053246	3.97654413	-3.49689471
C	3.76638468	3.25792278	-1.45394013
H	4.09144739	3.04817118	-0.42711079
H	4.50364695	2.81717370	-2.13757723
H	3.78235611	4.34643378	-1.59559234
C	0.24613279	2.84279761	2.92835447
H	0.67139746	1.83250325	2.97552466
C	1.03006816	3.73602063	3.89386776
H	0.99151466	3.32782146	4.91042301
H	2.08441886	3.83482489	3.60698848
H	0.60715332	4.74834464	3.92467843
C	-1.20873489	2.73203736	3.38041504
H	-1.25389777	2.29356860	4.38396575
H	-1.69882574	3.71296057	3.42821807
H	-1.79307588	2.08970372	2.71111237

INT2 (M = Mo): E(RM06) = -4031.86190066

Ge	-0.59630152	-0.34464620	-1.26787201
C	1.89507372	1.83658855	-0.84334573
O	1.69031964	2.87187557	-1.30911146
C	3.71440193	1.39369202	0.87674493
O	4.37639211	2.15017658	1.43930870
C	1.82257935	-0.12373116	1.75635034
O	1.41636603	-0.23711679	2.83168594
C	-1.79116965	0.12654709	0.34955506
N	-2.21940228	1.30379319	0.86203083
C	-3.20257956	1.08587587	1.80878353
H	-3.67962723	1.90616223	2.32712339
C	-3.38969908	-0.25699783	1.88463155
H	-4.06408598	-0.85841419	2.47872001
N	-2.52082996	-0.82582043	0.97674071
C	-1.79711219	2.61814549	0.43647712
C	-0.92140575	3.33692778	1.26092706
C	-0.56490605	4.62039764	0.83887794
H	0.11129235	5.21577656	1.44840049
C	-1.05293636	5.14458339	-0.34945058
H	-0.75866128	6.14387904	-0.66079175
C	-1.91464752	4.39935192	-1.14671944
H	-2.28472096	4.82621593	-2.07576995
C	-2.31103043	3.11540788	-0.77385701
C	-2.54777803	-2.21353441	0.58220967
C	-3.41108163	-2.56501177	-0.46823977
C	-3.39933200	-3.89853094	-0.87767415
H	-4.05011643	-4.21431190	-1.69097347
C	-2.57196443	-4.82936699	-0.26039818
H	-2.57983618	-5.86431042	-0.59399436
C	-1.74615842	-4.44867281	0.79013197
H	-1.12075326	-5.19545313	1.27569732
C	-1.71325633	-3.12607579	1.23873888
C	3.82633162	-1.98425230	-0.25580507
C	4.67679517	-0.86388637	-0.52619753
C	4.22019995	-0.22317255	-1.73528939
C	3.05993541	-0.92248986	-2.17822736
C	2.83640025	-2.03245789	-1.27351960
H	0.79661350	-0.54399625	-0.25775174
H	-0.39717916	1.16624659	-1.66345952
C	-0.84858574	-2.73491226	2.41750472
H	-0.82066125	-1.63640228	2.48201544
C	0.59049576	-3.21295531	2.25266591
H	1.03055693	-2.80158581	1.33372883
H	1.20332715	-2.88383313	3.10029171
H	0.65677641	-4.30771511	2.20315235
C	-1.45504953	-3.26035167	3.71973891
H	-2.48348503	-2.90542772	3.86015232
H	-1.48130014	-4.35778252	3.72551163
H	-0.86087087	-2.93279666	4.58078572
C	-4.34636735	-1.57446443	-1.13324799
H	-4.13856483	-0.56995468	-0.73508999
C	-5.80137388	-1.89794825	-0.79133074
H	-6.47386175	-1.14769936	-1.22424861
H	-6.09353000	-2.87769823	-1.19000413
H	-5.96590901	-1.91877235	0.29311946
C	-4.13815698	-1.51022774	-2.64546645
H	-4.77846676	-0.73439615	-3.08375736
H	-3.09441561	-1.28284980	-2.90314251
H	-4.39936655	-2.45902337	-3.13052142
C	-3.29496009	2.33307921	-1.62199882
H	-3.12752298	1.25903226	-1.44790515
C	-3.11969141	2.56496013	-3.11955952
H	-2.07995101	2.41638103	-3.43545223
H	-3.75128958	1.86701510	-3.68177237
H	-3.42424853	3.57633269	-3.41602815
C	-4.73211285	2.64118863	-1.19408736
H	-5.44521373	2.04711940	-1.78026541
H	-4.90216990	2.42255382	-0.13224075
H	-4.96252723	3.70212014	-1.35727264
C	-0.40134136	2.79194303	2.57549867
H	-0.51297674	1.69559893	2.56519391
C	1.07932030	3.09927228	2.78604666
H	1.46347019	2.54297490	3.64907969
H	1.67392029	2.82739288	1.90576248
H	1.24938566	4.16403406	2.98683355
C	-1.21344362	3.34410802	3.75001970
H	-0.84616175	2.93504445	4.69862740
H	-1.12296477	4.43715450	3.79680377
H	-2.28107073	3.10766600	3.67114098
C	5.95847629	-0.58810534	0.18825727
H	6.73156403	-1.28449881	-0.16244923
H	6.32743132	0.42623641	0.00828270
H	5.86365519	-0.72376810	1.27122665
C	4.90657805	0.89310508	-2.44958803
H	5.66527558	0.49886316	-3.13831874
H	4.20417017	1.48950503	-3.04137087
H	5.41436349	1.57383544	-1.75781931
C	2.32102961	-0.69452099	-3.45534097
H	2.76293215	-1.28524853	-4.26880621
H	1.26833228	-0.99263317	-3.37585074
H	2.34656098	0.35817590	-3.75772294
C	1.84039348	-3.11705748	-1.49676665
H	1.71628043	-3.75305313	-0.61418544
H	0.85035337	-2.72781949	-1.76992912
H	2.17545647	-3.75695858	-2.32390805
C	4.05539780	-2.97688225	0.83520333
H	4.28443141	-2.48918798	1.78974776
H	3.18529198	-3.62089739	0.99377314
H	4.90657638	-3.62530380	0.58798363
Mo	2.56639364	0.06696324	-0.11596265

3 (M = Mo): E(RM06) = -4032.13509715

Mo	-2.36456554	-0.39721569	-0.07298869

Ge	0.18376773	-0.45750086	-0.77216899
C	4.07262146	-0.57022734	-1.90334286
H	3.62776087	0.35272777	-1.50182397
C	3.45882197	-0.79644077	-3.28411032
H	3.93976986	-1.63083130	-3.80968022
H	3.58918679	0.09900749	-3.90537543
H	2.38428072	-1.01307649	-3.22103345
C	5.58002809	-0.33336607	-2.00514024
H	6.02487994	-0.12986728	-1.02317568
H	5.79144991	0.52127356	-2.65905615
H	6.08980041	-1.20983016	-2.42495641
C	0.84683259	2.80956769	0.38008974
C	0.04433153	3.26030211	1.43936140
C	-0.80680598	4.33356410	1.16798701
H	-1.45563812	4.71429284	1.95404877
C	-0.82690269	4.93175131	-0.08637327
H	-1.49329370	5.77056159	-0.27242572
C	-0.00040175	4.47075826	-1.10399919
H	-0.03386675	4.95075966	-2.07930426
C	0.85736231	3.38969609	-0.89963067
C	0.14628677	2.68707135	2.83909432
H	0.62679206	1.69788269	2.77671591
C	-1.20861643	2.48978824	3.51197207
H	-1.69881594	3.44638488	3.73070836
H	-1.07653446	1.96711354	4.46615473
H	-1.89258211	1.89694048	2.89690947
C	1.02193087	3.59197032	3.71190954
H	2.02377695	3.74267679	3.29272669
H	1.13584284	3.16513543	4.71525128
H	0.56108482	4.58254667	3.81829390
C	1.79304059	2.91723833	-1.99638967
H	2.03641682	1.86090622	-1.81052917
C	1.17403005	2.98129777	-3.38954249
H	0.19605348	2.48697885	-3.42590937
H	1.83571435	2.48579904	-4.11039029
H	1.04455361	4.01519495	-3.73319483
C	3.10710837	3.70106136	-1.95773112
H	2.92742812	4.76689856	-2.15007020
H	3.79630630	3.33084028	-2.72801169
H	3.61106401	3.61747869	-0.98650529
O	-1.95037501	2.14646543	-1.89925609
O	-4.11987117	1.63985888	1.59544404
O	-0.67088271	-0.65376116	2.58418854
N	2.79737066	-0.16173435	0.65139225
N	1.78181935	1.73876939	0.64195681
C	-2.44135786	-2.32159975	-1.50964344
C	-3.43698974	-1.39079185	-1.94496282
C	-4.38618239	-1.23064554	-0.88870745
C	-3.96588379	-2.05583514	0.21697494
C	-2.75908971	-2.72171034	-0.16950694
C	-1.41369791	-2.94600126	-2.39291835
H	-0.59345441	-3.39886461	-1.82722822
H	-0.98217905	-2.23381964	-3.10696182
H	-1.88065156	-3.74518796	-2.98440626
C	-3.52611505	-0.80367121	-3.31453466
H	-4.00414554	-1.51000100	-4.00623155
H	-2.53549745	-0.56581089	-3.72024117
H	-4.11615133	0.11847507	-3.32522176
C	-5.66325455	-0.46149398	-0.98226789
H	-5.53962890	0.46732721	-1.55003623
H	-6.05526403	-0.19398416	0.00406290
H	-6.43178031	-1.05902714	-1.48940639
C	-4.72930932	-2.29962927	1.47751926
H	-5.34441541	-1.43861908	1.75901084
H	-4.06214494	-2.51450341	2.31930852
H	-5.40194148	-3.15960959	1.36111298
C	-2.04098908	-3.76765267	0.61666038
H	-2.13647369	-3.60321917	1.69581602
H	-0.97116367	-3.79131688	0.37563206
H	-2.44739251	-4.76412351	0.39817365
C	-2.04227994	1.26114130	-1.16131985
C	-3.44390598	0.91294107	1.00710940
C	-1.28151114	-0.50795666	1.60734995
C	1.65244196	0.44826909	0.27056800
C	3.64239178	0.74147293	1.26261015
H	4.61585392	0.44407800	1.62733598
C	3.00471804	1.93817024	1.25374408
H	3.30049326	2.91389089	1.61272298
C	3.18361008	-1.50917656	0.30930230
C	2.93059899	-2.53416473	1.22858767
C	3.28517447	-3.82720687	0.83849489
H	3.11107788	-4.65867839	1.51851169
C	3.85882156	-4.06833359	-0.40517234
H	4.12443703	-5.08412576	-0.68824917
C	4.10631749	-3.02063526	-1.28490648
H	4.56573963	-3.22611345	-2.25008540
C	3.77488214	-1.70775950	-0.94690415
C	2.33890615	-2.26533304	2.59570278
H	1.89386247	-1.25857906	2.59150808
C	1.22391107	-3.24344050	2.95261511
H	0.45484874	-3.27359914	2.17078063
H	0.74101374	-2.93770414	3.88761265
H	1.60443407	-4.26232950	3.09695602
C	3.43919551	-2.28775441	3.65905234
H	3.02496493	-2.05830124	4.64773996
H	3.90801567	-3.27906604	3.71185114
H	4.23170211	-1.55995735	3.44477636
H	0.74525565	-1.89675116	-0.72919883
H	0.47461776	0.09365093	-2.19414201

2-W (M = W): E(RM06) = -4031.65656241

W	-2.09257094	0.19840514	-0.01749291
Ge	0.27859291	-0.33047774	-1.00566794
C	3.51883063	-1.97181206	-1.76827286
H	3.47601070	-0.91819601	-1.45534535
C	3.06265920	-2.02891167	-3.22345910
H	3.21538839	-3.02303780	-3.66177612
H	3.64810297	-1.32171479	-3.82407833
H	2.00174830	-1.76654291	-3.32854083
C	4.97840427	-2.41774836	-1.64266711
H	5.33973765	-2.34820452	-0.60931301
H	5.62440077	-1.79341858	-2.27183416
H	5.09649721	-3.45998584	-1.96615207
C	2.03823323	2.52356211	0.24178610
C	1.51660752	3.35394401	1.24405939
C	1.14022746	4.64407952	0.86367829
H	0.72932268	5.32146415	1.60950853
C	1.28259409	5.07521565	-0.44841150
H	0.98369022	6.08424537	-0.72148704
C	1.79981479	4.22358815	-1.41741180
H	1.89536220	4.57587009	-2.44166688
C	2.18902357	2.92254705	-1.09910179
C	1.39228209	2.91594420	2.68816526
H	1.51651694	1.82418896	2.73344076
C	0.01741932	3.23383097	3.27082796
H	-0.13110761	4.31315602	3.39950880
H	-0.08990346	2.76915866	4.25755260
H	-0.79005858	2.85978665	2.63103753
C	2.49098095	3.55719296	3.53887662
H	3.49602583	3.31999352	3.16836559
H	2.42262997	3.21399572	4.57786201
H	2.39339338	4.65056311	3.54027000
C	2.79864066	2.02045229	-2.15572528
H	2.61622044	0.97393733	-1.86876902
C	2.17168415	2.20124905	-3.53523349
H	1.07534137	2.15714085	-3.49367157
H	2.52157160	1.41006015	-4.20918486
H	2.45276901	3.15830721	-3.99177321
C	4.31610264	2.20503418	-2.21692820
H	4.56855245	3.23088274	-2.51572692
H	4.75733421	1.51963259	-2.95249461
H	4.79222744	2.01235999	-1.24698767
O	-1.19093953	2.70386002	-1.69009570
O	-3.36723026	2.32949724	1.93759693

O	-0.26220924	0.02597656	2.55411679
N	2.58784195	-0.95171110	0.76590394
N	2.47436764	1.19657687	0.61130166
C	-3.04988696	-0.70173033	-1.97866773
C	-4.08698584	-0.08190492	-1.20533638
C	-4.22367869	-0.82559029	0.02073786
C	-3.26436128	-1.87970578	0.01333643
C	-2.54807966	-1.81882659	-1.23041616
C	-2.66249388	-0.33778842	-3.37473159
H	-1.60760840	-0.55612247	-3.58073233
H	-2.82149907	0.72707787	-3.57459179
H	-3.26084745	-0.90388899	-4.10001433
C	-4.96639573	1.03855442	-1.65760141
H	-5.82793800	0.65346049	-2.21834231
H	-4.43224792	1.73626705	-2.31149287
H	-5.35607367	1.61736217	-0.81340773
C	-5.26807018	-0.61666773	1.06699529
H	-5.63933918	0.41290271	1.08119132
H	-4.89258100	-0.84717450	2.06983993
H	-6.12759573	-1.27224626	0.87650037
C	-3.14004702	-2.96389846	1.03104706
H	-3.35117922	-2.60175043	2.04327867
H	-2.13428644	-3.40088954	1.03518846
H	-3.84953020	-3.77465914	0.81598357
C	-1.63353214	-2.88455956	-1.74170499
H	-0.98409530	-3.29233197	-0.95492524
H	-0.99164023	-2.53392816	-2.55771320
H	-2.22904236	-3.71827364	-2.13748033
C	-1.46565013	1.80217257	-1.00821849
C	-2.86755294	1.56603609	1.23026688
C	-0.88447313	0.06220519	1.56988844
C	1.80927958	0.06141767	0.32638338
C	3.74196945	-0.45313561	1.34613476
H	4.49599439	-1.10252185	1.76895494
C	3.67141096	0.89829218	1.24570676
H	4.35171875	1.67752295	1.56115950
C	2.28369858	-2.32702770	0.44893460
C	1.61454147	-3.11852655	1.39599053
C	1.24586486	-4.40567495	0.99475874
H	0.72562776	-5.05484793	1.69637754
C	1.55328712	-4.87757429	-0.27633976
H	1.25741889	-5.88386304	-0.56424662
C	2.26396829	-4.08549089	-1.17168824
H	2.52758838	-4.48345313	-2.14944060
C	2.65451614	-2.79004149	-0.82941007
C	1.40550398	-2.66286219	2.82347892
H	1.48692450	-1.56841473	2.85497252
C	0.03544402	-3.03130459	3.38206666
H	-0.77456280	-2.67592649	2.73412525
H	-0.10142214	-2.57002196	4.36649558
H	-0.07763855	-4.11529124	3.51190521
C	2.51428756	-3.24580152	3.70549020
H	2.41152914	-2.88834297	4.73664883
H	2.46290465	-4.34239148	3.72188946
H	3.51407445	-2.96972567	3.34789322

INT1 (M = W): E(RM06) = -4032.82886423

Ge	-0.37129611	-0.59385648	-0.93644711
C	1.58093026	1.25475500	-1.37560739
O	1.39115585	2.02026197	-2.23246650
C	2.92713160	1.39631128	0.86218436
O	3.47237858	2.27007735	1.38521602
C	0.89883384	-0.01944523	1.52695677
O	0.28905686	0.04101565	2.51843851
C	-1.76745918	0.34799340	0.29137515
N	-2.08251889	1.61408654	0.63854345
C	-3.27700757	1.62837595	1.34228537
H	-3.70655047	2.55297690	1.70225617
C	-3.70795234	0.34533204	1.42664898
H	-4.58266610	-0.09333899	1.88705495
N	-2.77247586	-0.42431162	0.76241532
C	-1.34252036	2.80409952	0.28088637
C	-0.68356346	3.50603962	1.30208972
C	0.01515543	4.65769723	0.92987761
H	0.54751978	5.22691035	1.68901467
C	0.03663151	5.08764345	-0.38831221
H	0.58804707	5.98554805	-0.65633393
C	-0.64841605	4.38123018	-1.37076776
H	-0.61550227	4.73694378	-2.39637033
C	-1.35729837	3.21996645	-1.06585585
C	-2.89501987	-1.82894433	0.45572871
C	-3.46575567	-2.17198334	-0.78539818
C	-3.53726395	-3.52954737	-1.09327137
H	-3.96678335	-3.84080241	-2.04325418
C	-3.06929413	-4.49187455	-0.20351788
H	-3.13795483	-5.54545191	-0.46410788
C	-2.52809528	-4.11799466	1.01944657
H	-2.18285436	-4.88494780	1.71042750
C	-2.42843704	-2.77152341	1.38268509
C	2.38221231	-2.44975928	-0.67169619
C	3.23432734	-2.17645788	0.45313154
C	4.20238142	-1.21037822	0.04571589
C	3.94549873	-0.85760501	-1.32056077
C	2.81786732	-1.62744576	-1.76618613
H	-0.20358127	0.37358719	-4.49668413
H	-0.46615519	-0.15374347	-4.03823681
W	2.08052661	-0.16127057	-0.07754847
C	-4.01205913	-1.13198692	-1.74230678
H	-3.58008123	-0.15578847	-1.47783647
C	-3.63419652	-1.40347939	-3.19666622
H	-4.17609360	-2.26459551	-3.60732948
H	-3.89207587	-0.53655828	-3.81825396
H	-2.55906801	-1.59447713	-3.30702948
C	-5.53000391	-1.01310805	-1.58892944
H	-6.02211541	-1.95894229	-1.85029157
H	-5.81988890	-0.76031531	-0.56147619
H	-5.92290886	-0.23382863	-2.25353256
C	-1.88588307	-2.38199295	2.74065639
H	-1.75642785	-1.29118426	2.76846696
C	-0.51418694	-2.99723726	3.00264344
H	0.19222241	-2.72688040	2.20663373
H	-0.10428977	-2.62685037	3.94952205
H	-0.55885855	-4.09237665	3.06390338
C	-2.87948728	-2.76460515	3.83883457
H	-3.86135322	-2.30318225	3.67564768
H	-3.02800870	-3.85145122	3.87727975
H	-2.51081125	-2.44200897	4.81937295
C	-2.13660546	2.47912608	-2.14201181
H	-1.94342364	1.39552779	-2.03045128
C	-3.64538318	2.69758646	-1.98605619
H	-4.03255424	2.37137468	-1.01374962
H	-3.88207982	3.76354463	-2.10048492
H	-4.19230076	2.14850843	-2.76408789
C	-1.72462864	2.84349770	-3.56342290
H	-2.22650519	2.17304642	-4.27127190
H	-2.02965723	3.86634795	-3.81943987
H	-0.64353761	2.75634302	-3.71830864
C	-0.75186786	3.10755563	2.76326269
H	-1.12946043	2.07758484	2.82930933
C	0.61258181	3.12433614	3.44848454
H	0.52449364	2.71200512	4.46022395
H	1.35277782	2.52684444	2.90688067
H	1.00916103	4.14251514	3.54522842
C	-1.71869686	4.02657517	3.51588480
H	-1.81263760	3.71183773	4.56192387
H	-1.35262383	5.06136975	3.50976485
H	-2.72226981	4.03777736	3.07287479
C	2.31292251	-1.68558105	-3.17072334
H	2.91763448	-2.38322925	-3.76430531
H	1.27354686	-2.02955518	-3.22376079
H	2.35960844	-0.70703266	-3.66120581
C	1.37468757	-3.55211438	-0.74420952
H	0.82404467	-3.67339119	0.19700116
H	0.63689858	-3.39609200	-1.53941380

H	1.87855975	-4.50469329	-0.95453794
C	3.23242224	-2.89522773	1.76254401
H	2.28806232	-3.42005383	1.93722109
H	4.03325649	-3.64630762	1.78715733
H	3.39418314	-2.21510167	2.60638974
C	5.35394986	-0.74528728	0.87358944
H	6.18778007	-1.45387575	0.78581208
H	5.72236767	0.23520286	0.55521360
H	5.09283857	-0.67475380	1.93500972
C	4.78657212	0.03872261	-2.16983064
H	5.60426621	-0.52579473	-2.63667226
H	4.20540668	0.50111194	-2.97464776
H	5.23700048	0.84827384	-1.58532518

TS1 (M = W): E(RM06) = -4032.79368434

Ge	0.43516618	-0.06361232	-1.22891943
C	-1.70649045	-2.45512882	-0.92084124
O	-1.50650161	-3.45527130	-1.47272825
C	-2.92036300	-1.92905100	1.26014854
O	-3.41283700	-2.63017976	2.03620479
C	-0.99373544	-0.31979186	1.51266985
O	-0.36299479	-0.13516319	2.47006090
C	1.78326768	0.25938150	0.33337759
N	2.70985364	-0.58935142	0.83823127
C	3.57221429	0.08095419	1.68703670
H	4.38396504	-0.43163241	2.18479304
C	3.18322964	1.37805229	1.69650373
H	3.57913555	2.24282437	2.21048571
N	2.08599935	1.47272939	0.85576365
C	2.94346812	-1.94052740	0.38458633
C	2.39352317	-3.00977025	1.10236426
C	2.60160414	-4.29050940	0.58457202
H	2.18677390	-5.15117305	1.10563100
C	3.33088612	-4.48323569	-0.58215918
H	3.47398733	-5.48896198	-0.96969133
C	3.89233672	-3.39967932	-1.24846484
H	4.47888579	-3.56858853	-2.14973860
C	3.71570145	-2.09839563	-0.77831203
C	1.44053043	2.71823603	0.51462607
C	1.67284271	3.25231535	-0.77008951
C	1.04060987	4.45734224	-1.07906212
H	1.18789171	4.90247797	-2.06016576
C	0.24385116	5.11264742	-0.14597424
H	-0.23109446	6.05527948	-0.40789315
C	0.07834128	4.58428441	1.12621602
H	-0.52073154	5.12503590	1.85671504
C	0.67531714	3.37379838	1.49322266
C	-3.20392410	-0.03700251	-2.09352752
C	-2.67076172	1.18136456	-1.55698817
C	-3.27914834	1.41705125	-0.28809201
C	-4.21947570	0.36745743	-0.04363248
C	-4.18548955	-0.53256361	-1.16759370
H	-0.04624426	-1.57836413	-0.42956882
H	0.85978336	-1.68547527	-1.08620839
W	-2.13965626	-0.69618851	-0.09521572
C	-5.10486042	-1.68734326	-1.39913060
H	-6.00354674	-1.35814918	-1.93641710
H	-4.63266831	-2.47354701	-1.99764088
H	-5.43303202	-2.14463727	-0.45992197
C	-5.18788325	0.32823463	1.09333701
H	-6.02491545	1.01147319	0.90018034
H	-5.60926090	-0.67068931	1.24195679
H	-4.72248630	0.63230794	2.03750967
C	-3.12057103	2.65787881	0.52224682
H	-3.74669425	3.45888754	0.10501743
H	-3.43047459	2.50966952	1.56182471
H	-2.08523219	3.01901613	0.52401848
C	-1.82898620	2.16598088	-2.29341820
H	-2.48291606	2.92090538	-2.75242928
H	-1.12995187	2.69845150	-1.63578387
H	-1.25355578	1.70001648	-3.10013504
C	-2.92180486	-0.57679918	-3.45747700
H	-3.48641921	-0.01866372	-4.21555867
H	-1.85921452	-0.50133421	-3.71664845
H	-3.20703059	-1.62986961	-3.54541349
C	2.65397117	2.63253906	-1.74937453
H	2.66481939	1.54304921	-1.60245232
C	2.29676111	2.87069148	-3.21268140
H	2.94784393	2.26618173	-3.85499324
H	1.25792631	2.59496415	-3.43343697
H	2.44270694	3.91757595	-3.50674045
C	4.07061650	3.13679182	-1.45770071
H	4.38611944	2.91381324	-0.43074255
H	4.79077644	2.66759411	-2.14066733
H	4.12984031	4.22388954	-1.59894461
C	0.55862683	2.88513909	2.92383510
H	0.93369741	1.85501014	2.97619345
C	1.42066470	3.75877899	3.84050868
H	1.04207891	4.78867137	3.86866628
H	1.40634392	3.36974688	4.86520004
H	2.46560212	3.80868193	3.50995376
C	-0.87955383	2.85537665	3.43430188
H	-1.51728101	2.22266065	2.80788011
H	-0.90316388	2.44648864	4.45091156
H	-1.32376076	3.85825011	3.47487345
C	1.64279626	-2.82006851	2.40183861
H	1.45106025	-1.74727430	2.53881069
C	2.49815766	-3.29009288	3.58030484
H	1.97509821	-3.11646126	4.52799753
H	2.71202024	-4.36422715	3.50716714
H	3.46045048	-2.76454106	3.62067234
C	0.28975063	-3.52846608	2.39958675
H	-0.28187934	-3.25687405	3.29551735
H	-0.30778635	-3.25468962	1.51946031
H	0.40045367	-4.61987744	2.40045880
C	4.36917474	-0.93417810	-1.49662835
H	4.02115626	0.00268589	-1.03723650
C	3.99288523	-0.87393769	-2.97606384
H	2.90404277	-0.85800554	-3.11930077
H	4.41256517	0.03094125	-3.43499819
H	4.38956669	-1.73311343	-3.53092200
C	5.88745668	-0.97870841	-1.31817884
H	6.31403054	-1.87674397	-1.78269380
H	6.35543866	-0.10480178	-1.78786284
H	6.16899597	-0.98832424	-0.25792477

INT2 (M = W): E(RM06) = -4032.81746536

Ge	-0.78622038	-0.33816660	-1.26892003
C	1.72140293	1.81245400	-0.83045221
O	1.49634293	2.84568115	-1.29721303
C	3.50418236	1.39357923	0.89254468
O	4.16236557	2.15581150	1.45666825
C	1.66986961	-0.14768246	1.76330529
O	1.27346921	-0.27626718	2.84322070
C	-1.98540224	0.12673247	0.35005736
N	-2.41244876	1.30313373	0.86540270
C	-3.39736681	1.08414017	1.80941392
H	-3.87473494	1.90373746	2.32853572
C	-3.58648547	-0.25886110	1.88152978
H	-4.26331415	-0.86074819	2.47233368
N	-2.71665928	-0.82628921	0.97420116
C	-1.98773465	2.61847164	0.44497456
C	-1.10935108	3.33116688	1.27173410
C	-0.75107157	4.61585378	0.85505953
H	-0.07216439	5.20674626	1.46585876
C	-1.23995690	5.14646339	-0.33008974
H	-0.94404369	6.14656229	-0.63725456
C	-2.10382968	4.40658511	-1.12998693
H	-2.47363047	4.83828221	-2.05692016
C	-2.50180972	3.12155393	-0.76276320
C	-2.74245903	-2.21375049	0.57947728
C	-3.60254690	-2.56502164	-0.47350716
C	-3.58982819	-3.89866347	-0.88261566
H	-4.23787834	-4.21454003	-1.69808109

C	-2.76541647	-4.82959933	-0.26153862
H	-2.77281568	-5.86479610	-0.59435953
C	-1.94334739	-4.44892993	0.79201109
H	-1.32064102	-5.19608271	1.28041780
C	-1.91055819	-3.12601569	1.23978899
C	3.63071217	-1.99886606	-0.23790180
C	4.49083596	-0.88354059	-0.50302736
C	4.04151595	-0.23613508	-1.71023199
C	2.87173330	-0.92657737	-2.15906667
C	2.63928682	-2.03654721	-1.25783412
H	0.61538984	-0.49252011	-0.20576736
H	-0.59478201	1.17878724	-1.64680307
W	2.38869769	0.05655741	-0.10384971
C	-1.04954847	-2.73460473	2.42146479
H	-1.01438986	-1.63584582	2.48250307
C	0.38746925	-3.22130104	2.26579380
H	0.83378358	-2.81457068	1.34792185
H	0.99765789	-2.89167334	3.11494650
H	0.44833560	-4.31653386	2.22000468
C	-1.66488836	-3.25241480	3.72265243
H	-2.69237171	-2.89264283	3.85741864
H	-1.69610144	-4.34971736	3.73190077
H	-1.07312482	-2.92460769	4.58523491
C	-4.53480946	-1.57397603	-1.14217667
H	-4.33608649	-0.57130580	-0.73483181
C	-5.99229664	-1.90551489	-0.81942547
H	-6.66240597	-1.15373585	-1.25333609
H	-6.27702565	-2.88261546	-1.22975720
H	-6.16872640	-1.93620267	0.26291186
C	-4.30944579	-1.49837942	-2.65144821
H	-4.95010944	-0.72430700	-3.09236839
H	-3.26440117	-1.26191725	-2.89582351
H	-4.55742131	-2.44614427	-3.14542897
C	-3.48515113	2.34301244	-1.61515048
H	-3.31945445	1.26807688	-1.44366920
C	-3.30584159	2.57918857	-3.11160415
H	-2.26545199	2.43040512	-3.42532812
H	-3.93718223	1.88416744	-3.67775065
H	-3.60808953	3.59197121	-3.40554705
C	-4.92312460	2.65089588	-1.19008022
H	-5.63504104	2.05782835	-1.77879339
H	-5.09560541	2.43081565	-0.12893194
H	-5.15285974	3.71213850	-1.35217157
C	-0.58749286	2.77807373	2.58233702
H	-0.69326471	1.68106254	2.56305060
C	0.89127517	3.09163722	2.79680500
H	1.27936404	2.52513091	3.65140008
H	1.48722753	2.83736456	1.91243047
H	1.05426330	4.15436481	3.01383360
C	-1.40292544	3.31665897	3.76091498
H	-1.03193444	2.90395209	4.70649791
H	-1.31957489	4.40997513	3.81466095
H	-2.46903468	3.07357806	3.68179190
C	5.76435439	-0.60746339	0.22658257
H	6.54375813	-1.29988241	-0.11704004
H	6.13071904	0.40907676	0.05388379
H	5.65649950	-0.74433469	1.30811694
C	4.73567691	0.87583678	-2.42421356
H	5.47989934	0.47497258	-3.12463069
H	4.03474090	1.48675943	-3.00280694
H	5.26080661	1.54349941	-1.73282302
C	2.14862469	-0.69331069	-3.44538294
H	2.62548416	-1.25042941	-4.26251816
H	1.10529448	-1.02618162	-3.39056854
H	2.14445558	0.36645044	-3.72364505
C	1.63564376	-3.11529824	-1.48172128
H	1.50306575	-3.74650288	-0.59701504
H	0.64995120	-2.72012294	-1.75981212
H	1.97159503	-3.76086805	-2.30377734
C	3.84802485	-2.99472846	0.85260110
H	4.08162031	-2.51083325	1.80789020
H	2.97128953	-3.62989602	1.00946317
H	4.69270151	-3.65051202	0.60325918

3-W (M = W): E(RM06) = -4032.86931949

W	-2.19035481	-0.32602457	-0.08858552
Ge	0.35813556	-0.44188944	-0.80103169
C	4.25566614	-0.59358905	-1.89753232
H	3.81625508	0.33144674	-1.49486758
C	3.66057504	-0.80041440	-3.28922639
H	4.13774108	-1.63743256	-3.81407554
H	3.81388751	0.09784114	-3.90107189
H	2.58227494	-1.00140137	-3.24513031
C	5.76726370	-0.37427977	-1.97510598
H	6.20048302	-0.18579949	-0.98498136
H	5.99823036	0.48409964	-2.61745235
H	6.27216563	-1.25299389	-2.39618416
C	1.04144687	2.80336020	0.38970725
C	0.24371269	3.26383739	1.44847918
C	-0.61051830	4.33274788	1.16981111
H	-1.25807788	4.71882992	1.95432517
C	-0.63732049	4.91837495	-0.09013590
H	-1.30819169	5.75220475	-0.28239887
C	0.19136562	4.45399200	-1.10462179
H	0.15630697	4.92768682	-2.08286053
C	1.05362663	3.37804354	-0.89292104
C	0.35733738	2.71084133	2.85569392
H	0.84470369	1.72457853	2.80538603
C	-0.98976177	2.51403495	3.54429635
H	-1.49312321	3.46924849	3.73794704
H	-0.84140874	2.02297102	4.51282433
H	-1.66986985	1.89168331	2.95533500
C	1.23278652	3.63658810	3.70710622
H	2.22716407	3.79580819	3.27330426
H	1.36455058	3.22299498	4.71382567
H	0.76113733	4.62265713	3.80795924
C	2.00335650	2.91153016	-1.98058288
H	2.23375277	1.85033374	-1.80631877
C	1.41446910	3.00333021	-3.38483668
H	0.43268566	2.52050729	-3.45008529
H	2.08645525	2.51193418	-4.09890415
H	1.30394192	4.04384322	-3.71506715
C	3.32369059	3.68274028	-1.90612503
H	3.15638225	4.75251160	-2.08748174
H	4.02364172	3.31664693	-2.66870980
H	3.80949226	3.58207962	-0.92740015
O	-1.73314158	2.13154974	-2.01421490
O	-3.80231885	1.85039977	1.53176083
O	-0.47970414	-0.58684774	2.54595816
N	2.96496599	-0.18574434	0.64900547
N	1.96578586	1.72319355	0.65151868
C	-2.38952793	-2.20382303	-1.54408685
C	-3.46068980	-1.30477673	-1.84555304
C	-4.28362891	-1.18862935	-0.68199628
C	-3.71789557	-2.01482254	0.35285371
C	-2.53884213	-2.63688728	-0.18158631
C	-1.42981111	-2.75571011	-2.54541672
H	-0.54161327	-3.19062349	-2.07661658
H	-1.09834571	-1.99835794	-3.26594808
H	-1.91773702	-3.55438545	-3.11984606
C	-3.72936224	-0.69300190	-3.17993808
H	-4.31288257	-1.38108778	-3.80555250
H	-2.80174043	-0.46501475	-3.71697758
H	-4.29839212	0.23850788	-3.09418966
C	-5.58026236	-0.45080091	-0.61070008
H	-5.55130652	0.48218086	-1.18396185
H	-5.85205314	-0.19636453	0.41852147
H	-6.38946036	-1.06765616	-1.02193853
C	-4.32361665	-2.28655989	1.69120861
H	-4.89662442	-1.42934338	2.06016209
H	-3.56013495	-2.51593881	2.44229643
H	-5.00736770	-3.14391219	1.64013893
C	-1.71679648	-3.68561309	0.49120755
H	-1.74398989	-3.58156316	1.58157141

H	-0.66602355	-3.64563186	0.17799923
H	-2.09426256	-4.68688742	0.24585954
C	-1.84916315	1.27224851	-1.24621025
C	-3.17281633	1.07081319	0.95422400
C	-1.10045028	-0.42915452	1.57400415
C	1.82775580	0.43719779	0.26671097
C	3.81342580	0.70391610	1.27509596
H	4.78142264	0.39460004	1.64430932
C	3.18586267	1.90582681	1.27389248
H	3.48723320	2.87497470	1.64570896
C	3.33751991	-1.53518005	0.30117428
C	3.06772621	-2.56131927	1.21449591
C	3.40273943	-3.85731558	0.81716191
H	3.21462321	-4.69024120	1.49164737
C	3.97571671	-4.09934504	-0.42677657
H	4.22590309	-5.11741332	-0.71571000
C	4.24360579	-3.05011669	-1.29876227
H	4.70426884	-3.25679190	-2.26309340
C	3.93139110	-1.73431303	-0.95359664
C	2.48410482	-2.28802031	2.58428060
H	2.04557348	-1.27831597	2.58125061
C	1.36500832	-3.25790656	2.94955723
H	0.59076110	-3.28235583	2.17292549
H	0.89165450	-2.94843661	3.88823854
H	1.73894602	-4.27958971	3.09176907
C	3.59033613	-2.31810069	3.64147953
H	3.18342434	-2.08461310	4.63226648
H	4.05132176	-3.31322453	3.69220251
H	4.38786681	-1.59717049	3.42280493
H	0.89338922	-1.89268186	-0.76204873
H	0.67678122	0.10991836	-2.21544474
